# Compactness of Protein Folds Alters Disulfide‐Bond Reducibility by Three Orders of Magnitude: A Comprehensive Kinetic Case Study on the Reduction of Differently Sized Tryptophan Cage Model Proteins

**DOI:** 10.1002/cbic.201900470

**Published:** 2019-11-18

**Authors:** Dániel Horváth, Nóra Taricska, Ernő Keszei, Pál Stráner, Viktor Farkas, Gábor K. Tóth, András Perczel

**Affiliations:** ^1^ Laboratory of Structural Chemistry and Biology and MTA-ELTE Protein Modeling Research Group at the Institute of Chemistry Eötvös Loránd University 112, P. O. Box 32 1518 Budapest Hungary; ^2^ Chemical Kinetics Laboratory, Institute of Chemistry Eötvös Loránd University 112, P. O. Box 32 1518 Budapest Hungary; ^3^ Department of Medical Chemistry Faculty of General Medicine University of Szeged Szeged Dóm tér 8 H-6720 Szeged Hungary

**Keywords:** aggregation, kinetics, protein models, reduction, sulfur

## Abstract

A new approach to monitor disulfide‐bond reduction in the vicinity of aromatic cluster(s) has been derived by using the near‐UV range (*λ*=266–293 nm) of electronic circular dichroism (ECD) spectra. By combining the results from NMR and ECD spectroscopy, the 3D fold characteristics and associated reduction rate constants (*k*) of E19_SS, which is a highly thermostable, disulfide‐bond reinforced 39‐amino acid long exenatide mimetic, and its N‐terminally truncated derivatives have been determined under different experimental conditions. Single disulfide bond reduction of the E19_SS model (with an 18‐fold excess of tris(2‐carboxyethyl)phosphine, pH 7, 37 °C) takes hours, which is 20–30 times longer than that expected, and thus, would not reach completion by applying commonly used reduction protocols. It is found that structural, steric, and electrostatic factors influence the reduction rate, resulting in orders of magnitude differences in reduction half‐lives (900>*t*_1/2_>1 min) even for structurally similar, well‐folded derivatives of a small model protein.

## Graphical abstract

**Breaking bonds**: Structural, steric, and electrostatic factors influence the disulfide bond reduction rate, which results in three orders of magnitude differences in reduction half‐lives for N‐proximal truncated disulfide bond cyclized exenatide derivates. If the disulfide bond is near an aromatic cluster, near‐UV electronic circular dichroism spectral changes can be used to monitor reduction.

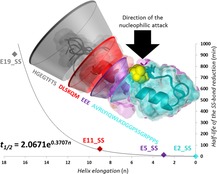

## Introduction

Structural disulfide (SS) bonds, which are stable in the harsh oxidative extracellular environment, maintain the native fold of proteins by fixing and protecting them from thermal fluctuation induced by elevated internal dynamics. The SS bond formation is perhaps the most fundamental post‐translational modification that stabilizes the 3D fold of globular proteins. The absence of regulated SS formation leads to diseases including diabetes,^1^ cancer,^2^ neurodegenerative conditions,^3^ and cardiovascular diseases.^4^ Non‐native SS bond pairing evokes backbone misfolding, which jeopardizes both function and bioactivity, although some proteins may present alternative SS states and still achieve similarly well‐folded forms.^5^ In protein evolution, the presence of SS bonds shows a significant correlation with the complexity of the organism.^6^ Approximately 50 % of all cysteine residues found in proteins form SS bonds,^7^ and thus, these cysteine residues become the most conserved among all amino acids, despite being added late to the genetic code during protein evolution.^8^ Due to the unique pairing pattern of cysteine residues, SS bonds stabilize the 3D fold of proteins unambiguously.^9^

Contrary to structural disulfides, redox‐active disulfides are highly dynamic, and their formation is reversible. The redox potential of the surrounding environment controls the regulation and cellular localization of these proteins.^10^ Intramolecular formation of these redox‐active disulfides is common for oxidoreductases (thioredoxin^11^ or glutaredoxin^12^ family) and allosteric disulfides,^13^, ^14^, ^15^ whereas an intermolecular SS linkage results in glutathionylated^16^ or cysteinylated^17^ small molecule–protein adducts. The redox potential and stability of the SS bond is highly dependent on several factors, such as the p*K*_a_ of the thiols (the standard p*K*_a_ is 8.5, but this can range from 3.5 to 12.8, depending on the local environment),^18^ the strain introduced by the SS bond of the protein structure, and the entropic cost of SS bond formation.^19^, ^20^ The Cys residues of an SS bond are typically distant in the primary sequence; 49 % of the SS‐bond‐forming cysteine residues are more than 25 residues apart from each other.^21^ The SS‐bond formation is thermodynamically more favorable if the cysteine residues are placed in spatial vicinity by the native fold itself before oxidation,^22^ otherwise—in the absence of chaperones assisting folding^23^—the protein precipitates. Adjacent cysteine residues oxidized to a SS bond are rare, although examples can be found among enzymes, receptors, and toxins.^24^, ^25^ The SS bond or SS bond pattern in prokaryotic proteins is formulated by ribosomal mRNA translation, followed by oxidation and post‐translational modifications catalyzed by various enzymes located in the periplasm (DsbC, DsbG, DsbD)^26^ or cytoplasm (DsbA, DsbB).^27^, ^28^ In eukaryotic species, this process is performed in specific cell organelles, such as the mitochondria (Mia40, ERV1), endoplasmic reticulum (PDI, ERO1, Erv2), and chloroplasts (PSI, PSII, LTO1, LQY1, CYO1).^29^

The SS bonds form the core of hundreds of proteins of known 3D structures. Hydrophobic and/or aromatic residues (e.g., Trp and Tyr) may condense around the SS bond and form the network of key interactions that determine the 3D structure of a large number of different proteins.^21^ In at least 50 % of protein families, this type of interaction is invariant. In dozens of proteins (e.g., tick anticoagulant peptide,^30^ phospholipase A2^31^) the SS unit(s) are reinforced by associated aromatic–aromatic interactions^32^, ^33^ and vice versa. For instance, 92Tyr of RNase‐A effectively shields the solvent‐exposed nearby SS bond (40Cys–95Cys) from reducing agents (RAs), and thus, helps to maintain the native fold of the protein.^34^

If SS bonds are reduced, the thiol groups of the free cysteine residues often adopt an ensemble of local conformers that also loosen the compactness of neighboring residues. In the era of manufacturing recombinant proteins (e.g., insulin), the SS bond cyclized peptides (e.g., vasopressin, oxytocin, desmopressin, octreotide)^35^ and human monoclonal IgG antibodies,^36^ which are produced on a large scale by the biopharmaceutical industry, it is vital to have reliable and fully tested methods for SS bond reduction.

In addition to β‐mercaptoethanol or 1,4‐dithio‐d‐threitol (DTT), more recently tris(2‐carboxyethyl)phosphine (TCEP) has become commonly used as a RA of SS bonds because it is chemically more stable, nonvolatile, odorless, and it reduces SS bonds more effectively, even at low pH.^37^, ^38^ TCEP is claimed to selectively and completely reduce water‐soluble alkyl disulfides over a wide pH range within a few minutes (<5 min).^39^ Some protocols recommend using 1–100 molar equivalents of TCEP relative to protein concentration.^40^, ^41^ The reduction time and appropriate temperature greatly depend on the nature of the protein, but, generally, elevated temperature and/or TCEP^42^ concentration and longer times make the reduction more complete, but these conditions also initiate a multitude of side reactions, which are poorly described, to date.

**Figure 1:**
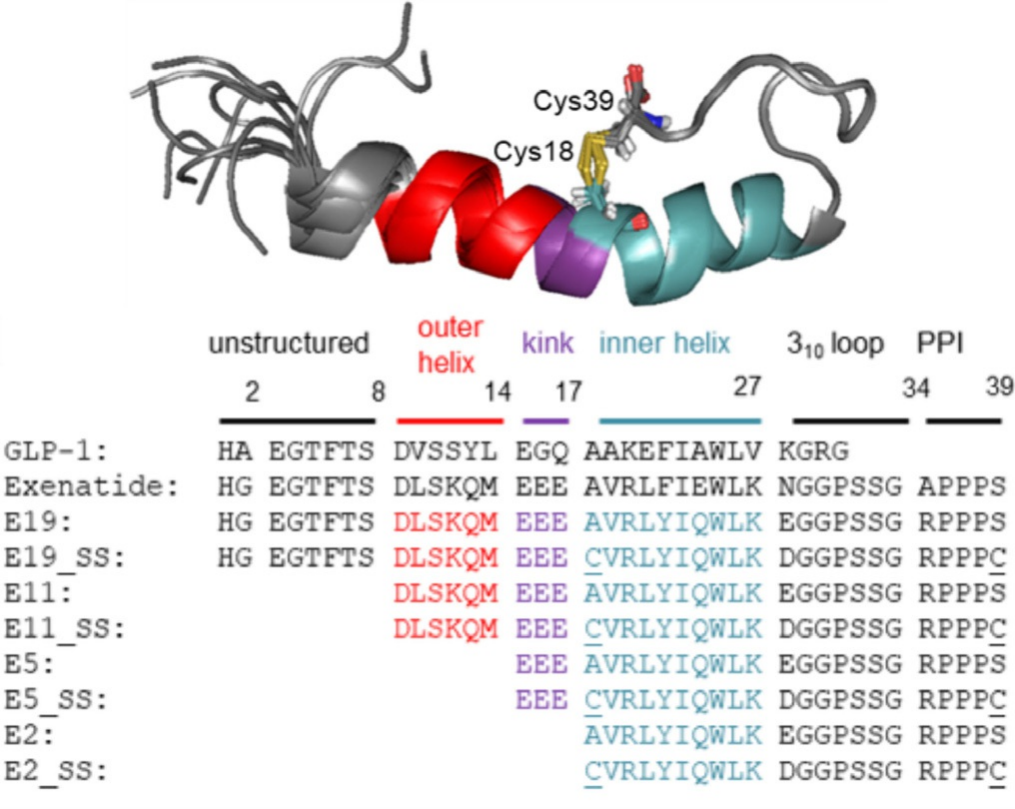
A) Structure ensemble of E19_SS, and B) amino acid sequences of GLP‐1; exenatide; parent E19 and its truncated derivatives E11, E5, and E2; their SS analogues E19_SS, E11_SS, E5_SS, and E2_SS; and their reduced 2SH analogues E19_2SH, E11_2SH, E5_2SH, and E2_2SH. The position of the SS bridge is highlighted by stick representation and underlined as C. The sequences of E19 is divided into six major parts: 1) 2–8 unstructured N terminus, 2) 9–14 outer helix, 3) 15–17 kink region, 4) 18–27 inner helix, 5) 28–34 3_10_ helix, and 6) 35–39 polyproline region. This apportionment of the sequence coincides with the truncation of the peptides.

Exendin‐4^43^ or exenatide^44^ (synthetic name), which as been used in clinical practice since 2005, is an incretin mimetic^45^ glucagon‐like peptide‐1 (GLP‐1) analogue, which is a 39‐residue peptide with complex physiological actions^46^ in multiple organs, used in the treatment of type 2 diabetes mellitus.^47^ Exenatide acts as an agonist of the GLP‐1 receptor^48^ (GLP‐1R). Its amphipathic helix binds to the extracellular domain of the GLP‐1R, the mainly unstructured N terminus activates the receptor,^49^ and the structure‐stabilizing Trp‐cage^50^, ^51^ fold is not directly involved in interactions to GLP‐1R.^52^, ^53^ We have synthesized and studied the 3D fold of several dozens of Trp‐cage folds, including analogues of exenatide, such as E19,^54^, ^55^ which is a 39 amino acid protein of comparable bioactivity, but improved water solubility. As a “natural tool” for enhancing the compactness of the 3D fold, we introduced two solventexposed Cys residues into E19, making E19_SS (Figure 1), and a loop from residues 18 to 39 in E19_A18C_S39C (E19_2SH). E19_2SH oxidized to E19_SS spontaneously with atmospheric O_2_ dissolved in water at room temperature. The SS bond of E19_SS extends the hydrophobic core of the native Trp fold in the spatial proximity of 22Tyr, which is surrounded by explicit negative charges (15Glu, 16Glu, 17Glu). Although E19_SS is small in size, (MW: 4334.9 g mol^−1^), its reduction takes several hours to reach equilibrium with >10 molar excess of TCEP in water at room temperature. Because the SS bond reduction time turned out to be significantly longer than that expected based on literature data and common laboratory practice, we launched a comparative study, including three designed and truncated analogues of E19_SS, namely, E11_SS, E5_SS, and E2_SS. Notably, the model systems thus created (Figure 1) oxidize spontaneously and rapidly adopt the Trp‐cage 3D fold.^56^ Moreover, the “loop size” created by the SS bond, in other words, the number of residues between the two reacting cysteine residues, is 20 amino acids long, which is close to the average value (≈17) observed in thousands of proteins.^57^

E11_SS was designed by removing the “HGEGTFTS” tail, which was the unstructured GLP‐1R‐activating N‐terminal eight residues of E19_SS. Shortening by an additional six residues removes the outer helical part of E19_SS, namely, the “HGEGTFTS‐DLSKQM” subunit,^56^ affording E5_SS. Although 14 residues shorter than that of E19_SS, E5_SS still adopts a compact Trp‐cage fold and comprises the entire interface for binding to GLP‐1R.^52^ Finally, in E2_SS, the entire N terminus preceding 18Cys of E19_SS was omitted, namely, “HGEGTFTS‐DLSKQ‐EEE” was cleaved, to give a folded protein with a fully exposed SS bond at its surface; this is considered to be a construct ready for a rapid SS bond reduction (Figure 1 **)**.

Herein, we discuss the structure and properties of both the oxidized and reduced forms of the four model proteins of different α‐helical lengths, in comparison with the parent (Cys‐free) miniproteins, and the kinetics of reduction. We introduce spectroscopic approaches that make the monitoring of the reduction progress fast and easy. The effect of the compactness of the protein fold, the accessibility, and the local explicit charges of the SS bond and the reagent type on reduction rate and the mechanism are also explained herein.

## Results and Discussion

### Three‐dimensional fold characterized by far‐UV electronic circular dichroism (FUV‐ECD) spectra

**Figure 2:**
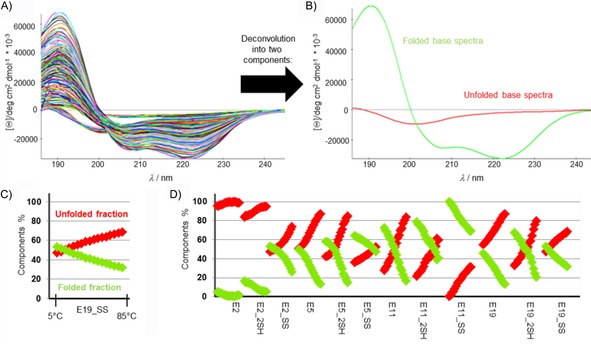
A) Temperature‐dependent FUV‐ECD spectra (204 in total) of the four primal peptides (E2, E5, E11, and E19) and their four reduced (_2SH) and four oxidized (_SS) variants. B) The two pure ECD curves were derived from the ensemble analysis of the 204 ECD spectra by using CCA+. Pure component 1 (red) represents that of the unfolded/U‐type, whereas component 2 (green) represents the folded/C‐type backbone structure. C) The associated relative propensities [%] of the two pure components at each measured temperature are given for E19_SS as an example, D) as well for each 204 spectra starting from E2 (at 5 °C) up to E19_SS (at 85 °C).

Circular dichroism (CD) spectroscopy is increasingly recognized as very sensitive indicator of protein conformation,^58^, ^59^ relying on a plethora of electronic transitions. The FUV‐ECD spectra of Trp‐cage proteins (e.g., Exenatide, E19, E19_SS) are typically the weighted sums of the C‐ (folded and highly helical) and U‐type (unfolded) base curves (Figure 2 A), as assigned and verified by means of NMR spectroscopy.^60^, ^61^ As the temperature increases, the shape of the FUV‐ECD spectra changes: those of the parent proteins—E2, E5, E11, and E19—acquire more and more U‐type characteristics, as they unfold gradually. The temperature‐dependent FUV‐ECD spectra for all four SS bond enforced model peptides were recorded between 5 and 85 °C (in steps of 5 °C, resulting in 17 spectra for each protein; Figure S1 in the Supporting Information). Aside from E2_SS, the SS‐bond‐containing mutants have similar FUV‐ECD spectra to that of their parent proteins at low temperatures. On the other hand, because the SS bond makes the 3D folds of SS variants more rigid, they preserve their C‐type characteristics better and delay unfolding, even at higher temperatures. Once the SS bond is reduced (see below for details), the spectral properties of the SH variants revert to those of the parent proteins. Their 3D‐scaffold compactness decreases as the temperature increases; this is less apparent in the case of E2 and E2_2SH because they both already present an ensemble of dynamic backbone structures at 5 °C.

Ensemble deconvolution^62^, ^63^ of the 204 (12×17) ECD spectra, *f*(*λ*, *T*), made the quantitative analysis of the relative abundance of secondary structural elements belonging to each peptide in each state possible because the pure ECD curves were successfully assigned.^60^, ^64^, ^65^, ^66^ The results in Figure 2 B indicate that 1) the SS bond stabilizes the less folded protein scaffolds more effectively, for example, whereas the difference at 4 °C between the E2 and E2_SS folded fraction is 48 %, the same difference between E5 and E5_SS is 14 %; in the case of E11 and E11_SS, it is only 28 %, and for E19 and E19_SS it is 7 % (Figure 2 D). 2) The ratio of the folded, helical components increases upon going from E2 to E5 and E11; however, the compact α‐helical content of E19_SS, E19_2SH, and E19 is lower than those of E11_SS, E11_2SH, and E11 because the unfolded eight‐residue‐long N‐terminal part elevates the overall backbone dynamics, which destabilizes the compact 3D fold. 3) All four reduced proteins (E2_2SH, E5_2SH, E11_2SH, E19_2SH) have a higher helix content (≈7–15 %) than that of the parent proteins over the entire temperature range. 4) The 3D folds stabilized by SS bonds are less sensitive to temperature (Figure 2 C, D).

### Three‐dimensional folds of proteins determined and characterized by NMR spectroscopy

The ensemble of the temperature‐dependent FUV‐ECD spectra confirms that SS bonds preserve the fold of the model proteins and increase thermostability. NMR spectroscopy analysis at 15 °C allowed further characterization of the 3D structures of each variant. Fold, chemical shift, and secondary chemical shift (SCS) information^67^ were derived from the appropriate 2D homonuclear NMR spectroscopy experiments (^1^H,^1^H COSY, ^1^H,^1^H TOCSY, and ^1^H,^1^H NOESY) at *T=*15 °C; the ensemble of the ten lowest energy structures were analyzed. This comprehensive analysis conducted at 15 °C provided the following useful structural descriptors: the root‐mean‐square deviation (RMSD) of the 3D structures, the average chemical‐shift deviation (CSD) of backbone Hα protons per residue ([∑CSDHα(i) ]/*i*) in the helical segment and the compactness of the Trp‐cage core by SCS sum of selected protons: CSD_cage_ (Table 1).

**Table 1:** Selected measures characterizing the degree of folding of the model protein (*T=*15 °C and pH 7).

		Degree of the fold by FUV‐ECD [%]^[a,b]^	Backbone RMSD [Å]^[c]^	CSD_cage_	[∑CSDHα(i) ]/*i*
E19	_SS	51.6	0.7	11.7	0.5
	_SH	65.8	0.7	11.1	0.6
	parent	43.7	1.2	10.9	0.5
E11	_SS	96.0	0.3	11.5	0.5
	_SH	76.0	0.3	11.0	0.6
	parent	70.0	1.5	10.9	0.5
E5	_SS	63.4	0.2	11.4	0.5
	_SH	42.8	0.1	10.4	0.5
	parent	51.5	1.6	10.3	0.4
E2	SS	51.8	0.1	11.3	0.5
	_SH	14.8	0.3	9.6	0.4
	parent	3.7	1.5	3.8	0.3

[a] *T=*15 °C, *c*_(protein)_=20–30 μm at pH≈7 (typical conditions applied for CD measurements). [b] Calculated % from the joint deconvolution (CCA+) of 204 *T*‐dependent FUV‐ECD spectra (Figure 2). [c] *T=*15 °C, *c*_(protein)_=0.8–1.8 mm at pH≈7 (typical conditions applied for ^1^H NMR spectroscopy measurements). [d] RMSD of all backbone atoms of the ten best structures. [e] Xf‐cage values^51^, ^55^ were used to correlate the fold of the protein. The following “H” atoms were involved in calculations: W25H*ϵ*1, L26Hα, G30Hα2, P31Hβ2, R35Hα, P37Hα, P37Hβ2, P38Hδ1, and P38Hδ2. [f] The average CSD of backbone Hα protons per residue.

**Figure 3:**
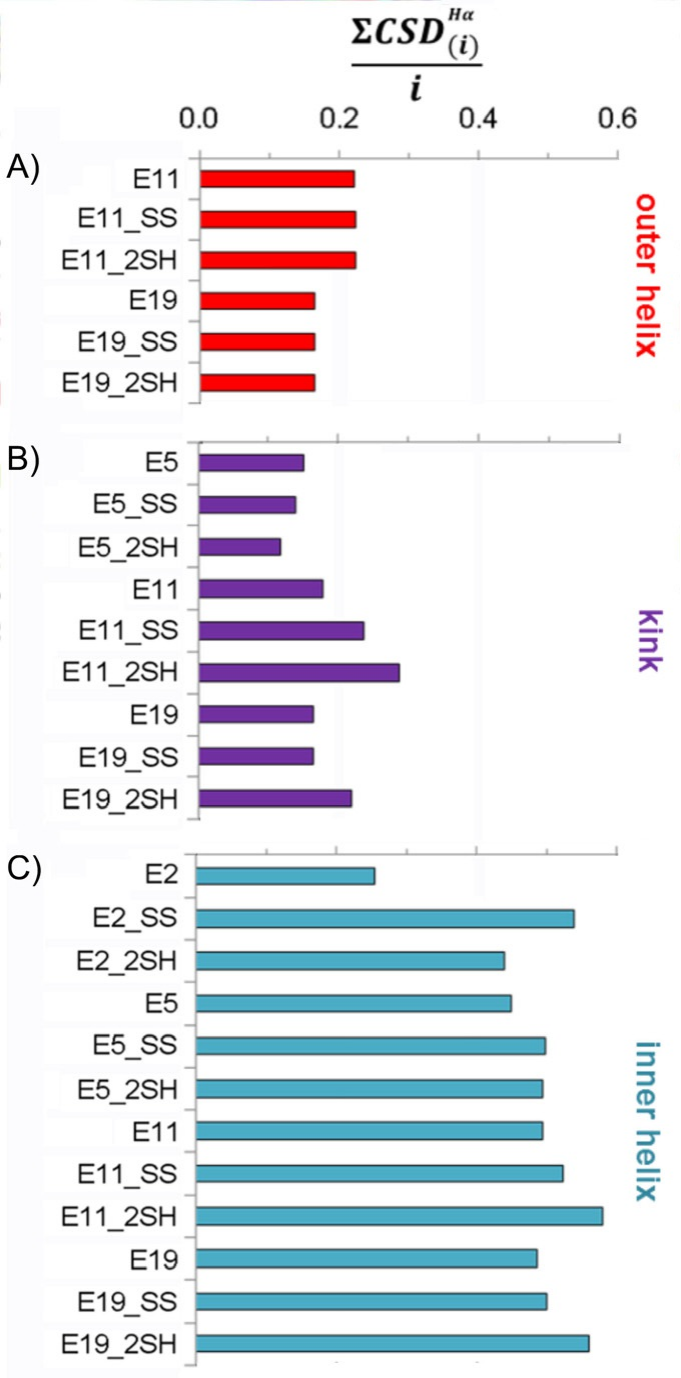
The average CSD of backbone Hα protons per residue ([∑CSDHα(i) ]/*i*) in the three different helical regions: A) outer helix (*i*=6), B) kink region (*i*=3), and C) inner helix (*i*=10). Higher residual values imply a more structured α‐helix.

A comparison of the helices of different lengths is more straightforward if the helical segment is divided into three parts: 1) the outer α‐helix, 2) the kink region in the vicinity of the SS bond, and 3) the inner α‐helix (Figure 3).

The outer helix compactness seems to be affected by the length of the α‐helix. Interestingly, this part of E11 variants is slightly more structured, in spite of being the terminal part (usually flexible and unstructured), as opposed to the outer helices of the E19 variants, where this helical segment is flanked (Figure 3 A). The above tendency is also true for the kink region, but here the presence and state (_SS or _2SH) of the SS bond are also differentiated (Figure 3 B). These distant helical parts have generally lower [∑CSDHα(i) ]/*i* values than those of the inner helix. The compactness of the inner helices is similar (expect for E2). Interestingly, reduced longer polypeptides show slightly increased [∑CSDHα(i) ]/*i* values, which may be the indicative of ring tension in the SS bond cyclized variants in these systems (Figure 3 C).

^1^H NMR spectroscopy studies also confirm that all model proteins, except E2, have a common, compact, and folded Trp‐cage core structure at *T=*15 °C (Tables 1 and S1 and Figure S2), regardless of the differently structured tails attached to them (Table 1). E2 is predominantly unfolded, even at low temperature (15 °C), but because the SS bond joins together the N and C termini of E2_SS, the hydrophobic core folds properly. Interestingly, even E2_2SH forms a more compact Trp cage than that of E2. In agreement with data reported in the literature, cysteine residues promote and stabilize α‐helices, if located at their N termini.^68^ The cage values of the longer reduced peptides are close to that of their oxidized counterparts (Table 1). NMR spectroscopy data reveal that a longer α‐helix results in a more structured Trp cage, in all cases studied.

In general, it seems that the core of the reduced (SH^−^) proteins is almost as well folded as those that are SS bonded. The following 3D fold compactness order was established: CSDSScage >CSD2SHcage >CSDparentcage , but the differences are small, aside from those of E2 (CSDE2cage =3.8)→E2_SS (CSDE2_SScage =11.3).

### Oxidized and reduced states defined by near‐UV (NUV) ECD data

**Figure 4:**
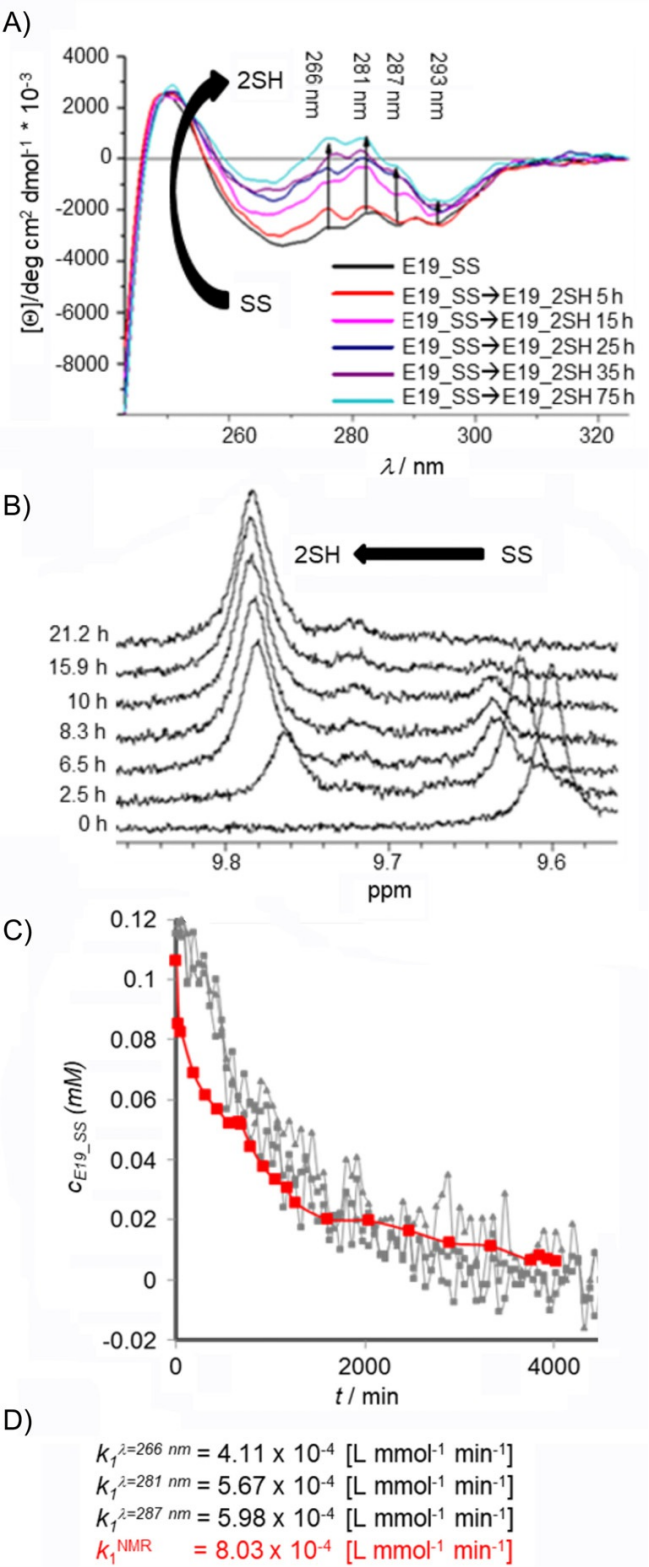
A) NUV‐ECD spectral changes measured for the reduction of E19_SS (≈0.113 mm E19_SS, pH  7, 15 °C, 18‐fold excess of TCEP) at four different wavelengths (*λ*=266, 281, 287, and 293 nm). No spectral changes were observed after about 55 h (3300 min). B) ^1^H NMR spectra of E19_SS→E19_2SH reduction (*c*≈0.115 mm, pH 7, 15 °C, 18‐fold excess of TCEP) in water. The chemical shift of the indole H*ϵ*1 of Trp25 was used to monitor reduction: H*ϵ*1 shifted upfield from *δ*=9.60 (SS) to 9.78 ppm (2SH during the reduction). Reaching steady state, the integral ratio of E19_2SH and E19_SS was found to be 92 to 8 %. C) Concentration change of E19_SS [mm] measured during reduction by different approaches plotted as a function of time. D) The calculated rate constants (see the discussion of modeling reduction kinetics).

As shown above, reduction does not have a dramatic effect on the tertiary structure content of the model systems at room temperature; thus, to detect reduction, NUV‐ECD spectra (instead of FUV) had to be used. The interpretation of the changes to the observed chiroptical properties of the Trp/Tyr/SS→Trp/Tyr/2SH (Figure 4 A) complex chromophore system is less straightforward because the assignment of “pure” NUV‐ECD spectra has not yet been completed. The conformation‐dependent fine structure of Tyr/Trp chromophores^60^ (260≤*λ*≤320 nm) comprises the ^1^Lb of Tyr (*λ*≈276 nm, with a shoulder at *λ*≈287 nm), ^1^Lb of Trp (*λ*≈281 and ≈293 nm), and ^1^La of Trp transitions appearing as superimposed broad bands. In addition, the SS bond may also contribute in form of a relatively weak but broad band with a maximum at *λ*≈260–270 nm. For the current proteins with SS bonds, a larger negative band was recorded (Figure S3). The bands of Trp and Tyr in the SS‐bond‐containing proteins shifted to the negative ellipticity range, which was not observed in the case of the parent proteins (E2, E5, E11, E19),^60^ for which the bands of these amino acids were detected in the positive range (except the Trp band at *λ*≈293 nm). The reduction kinetics of E19_SS→E19_2SH were monitored over time as the band intensities at *λ*≈266, 281, 287, and 295 nm increased from larger negative to smaller negative and/or positive values, similar to those of the parent proteins (Figures 4 A and S3). We were encouraged to use NUV‐ECD spectral changes to monitor SS to SH reduction in proteins if embedded in a suitable molecular environment such as that of the Trp cage motif.

Due to the acidic nature of TCEP, to avoid any pH shift, a phosphate buffer (50 mm, pH 7) was used to sustain near‐physiological pH. The groups of Han^39^ and Whitesides^37^ described the chemical instability of TCEP above pH 7 in 300–400 mm phosphate buffer. They found that the autoxidation of TCEP depended on how the reagent was stored (open air or in capped vials), whether the solution is stirred, and on the elapsed time (24≤*t*≤72 h) during storage. However, herein, we have monitored TCEP stability by means of ^31^P NMR spectroscopy in 50 mm phosphate buffer, and found no significant spectral changes connected to TCEP oxidation or degradation at room temperature over 14 days.

Reduction of the E19_SS protein was followed by recording NUV‐ECD spectra (≈0.113 mm E19_SS, pH 7, 15 °C, 18‐fold excess of TCEP, cell length=10 mm) at four different wavelengths (266, 281, 287, and 293 nm). Thus, by following band intensity changes of selected (one or more) ^1^Lb transitions of Tyr or Trp, we could monitor the redox state of the SS/SH groups and determine the “end point” as a steady state. Thus, if a suitable aromatic residue (Tyr, Trp, Phe) is coupled to the SS bond as a chromophore, it enables its reduction/oxidation state to be monitored, even if the molecular system shows no coupled backbone conformational changes (CSDE19_SScage =11.66; CSDE19_2SHcage =11.07). The measured absorbance was converted into concentration by using Equation (1):

(1) $$c\left(t\right)=\frac{A{}_{\infty }-A}{A{}_{\infty }-A{}_{0}}\left[\mathrm{SS}\right]{}_{0}$$

Steady state was reached conclusively after about 55 h. We determined the rate constant, *k*_1_, at each wavelength by parameter estimation to be kλ=266nm1 =4.11×10^−4^ L mmol^−1^ min^−1^, kλ=281nm1 =5.67×10^−4^ L mmol^−1^ min^−1^, and kλ=287nm1 =5.98×10^−4^ L mmol^−1^ min^−1^ (Figures 4 C and S4). The deviations of the fitted model from the measured data at *λ*=293 nm were remarkably large; therefore, parameter estimation was not performed on this dataset. NUV‐ECD monitoring enables one to observe the clean and clear changes in the spectra, but it does not make it possible to extract the absolute value of the concentration, [SS]_∞_, at the end point of the reaction. Based only on the intensity of the molar ellipticity, it cannot be decided if reduction is fully completed or not. To ascertain the absolute values of the concentrations in the redox system, reduction was repeated under the same conditions in NMR tubes with a diameter (Ø) of 5 mm (≈0.113 mm E19_SS, pH 7, 15 °C, 18‐fold TCEP) by recording ^1^H NMR resonances (Figure 4 B). By using both SS and SH state integrals of the signals at selected resonance frequencies (e.g., H*ϵ* ^Trp^), ^1^H NMR spectroscopy driven quantitative analysis of the reduction was performed (Figure 3 E) and the rate constant was determined to be kNMR1 =8.03×10^−4^ L mmol^−1^ min^−1^. Although 18‐fold excess of TCEP was used, ^1^H NMR spectroscopy data showed that, at steady state, ∂[E19_SS]/∂*t≈*0 and ∂[E19_2SH]/∂*t≈*0, reduction was incomplete and about 8 % of E19_SS remained oxidized. A comparison of the calculated reaction rates of the two methods (NMR and CD spectroscopy) shows that not only are the orders of magnitudes the same, but the values are also quite similar. The reduction rate of NUV‐ECD measured at *λ*=287 nm is closest to that of kNMR1 (Figure 4 C). Monitoring the intensity of the molar ellipticity by NUV is a fast and efficient method to define the end of the reaction. It also provides an approximate value of the reduction rate if the conversion is close to completion. Based on ^1^H NMR spectroscopy integrals, it is possible to determine the rate of the conversion and obtain evidence for the reversibility of the redox system. Taking into account incomplete conversion, despite the presence of the 18‐fold excess of RA, the role of dissolved oxygen and reoxidation should also be included in the kinetic mechanism.

### Concept of the reversible redox system

**Figure 5:**
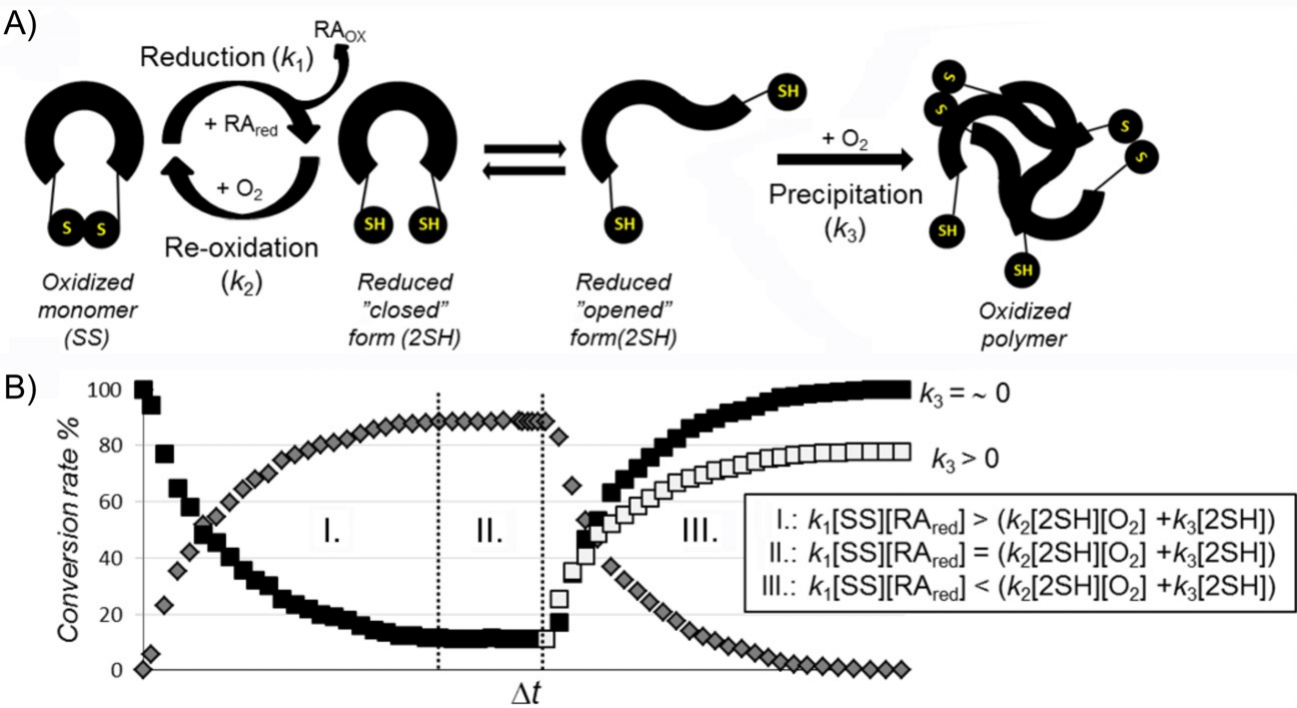
A) A schematic illustration of the redox cycle. The oxidized state (SS) in the presence of RA (e.g., TCEP) becomes reduced (2SH), in which open and closed conformers are present in equilibrium. In the case of the closed conformer, in which the −SH groups are closely fixed to each other, intramolecular reoxidation can occur in the presence of O_2_, whereas the open conformer is more likely to aggregate due to intermolecular interactions. B) Three stages of the theoretical redox setups provide the state at which reduction dominates the overall process (I), a steady state (II), and a state (III) in which excess dissolved O_2_ and the absence of RA lead to oxidation back to the reduced state. The black square denotes the relative concentration of the oxidized form; gray diamonds represent the reduced form. If precipitation occurs (*k*_3_>0), then at the end point of the redox cycle the soluble protein concentration has decreased relative to the initial one.

Physiological solutions contain dissolved O_2_ from the air, and thus, Cys‐SH groups of any protein might oxidize spontaneously to form the SS bond(s). The apparent rate constant depends on several microequilibrium constants, which are explicitly not elaborated herein.^69^ However, it certainly depends on the width of the conformational space of the reduced molecular fold. Furthermore, the concentration of dissolved O_2_ (and thus, *T* and *p*), the diffusion rate of TCEP, and the protein concentration are all rate‐influencing factors. Because our model protein forms a coupled reaction cycle, once E19_2SH is reduced by excess TCEP, E19_SS will be instantaneously reoxidized by dissolved O_2_ (Figure 5). Before exploring the mechanism of these redox‐cycle‐related electron‐transfer processes, it should be noted that, at a macroscopic level, these coupled cycles remain hidden, as steady state (∑∂ξ/∂*t*=0) is reached. Reduction concludes in a “normal way” if all dissolved O_2_ is consumed; however, if the concentration of the RA declines faster than that of O_2_, then oxidation will dominate the process and spectral properties will change accordingly (Figure 5). It is hard to a priori predict the end point of the latter process because, unlike the oxidized fold of a protein, the reduced one could have a multitude of backbone conformers in exchange at various timescales (e.g., μs to ms). Among these 3D folds of the reduced state, the “closed‐SH” forms (Figure S2 II), in which both the C and N termini are close to each other, lead only to intramolecular reoxidization. However, if “open‐SH” backbone forms become highly populated (e.g., as is the case of E2_2SH; Figure S2/XIII), then intermolecular oxidation will be more prevalent, giving rise to oligo‐ and polymer formation (see below).

**Figure 6:**
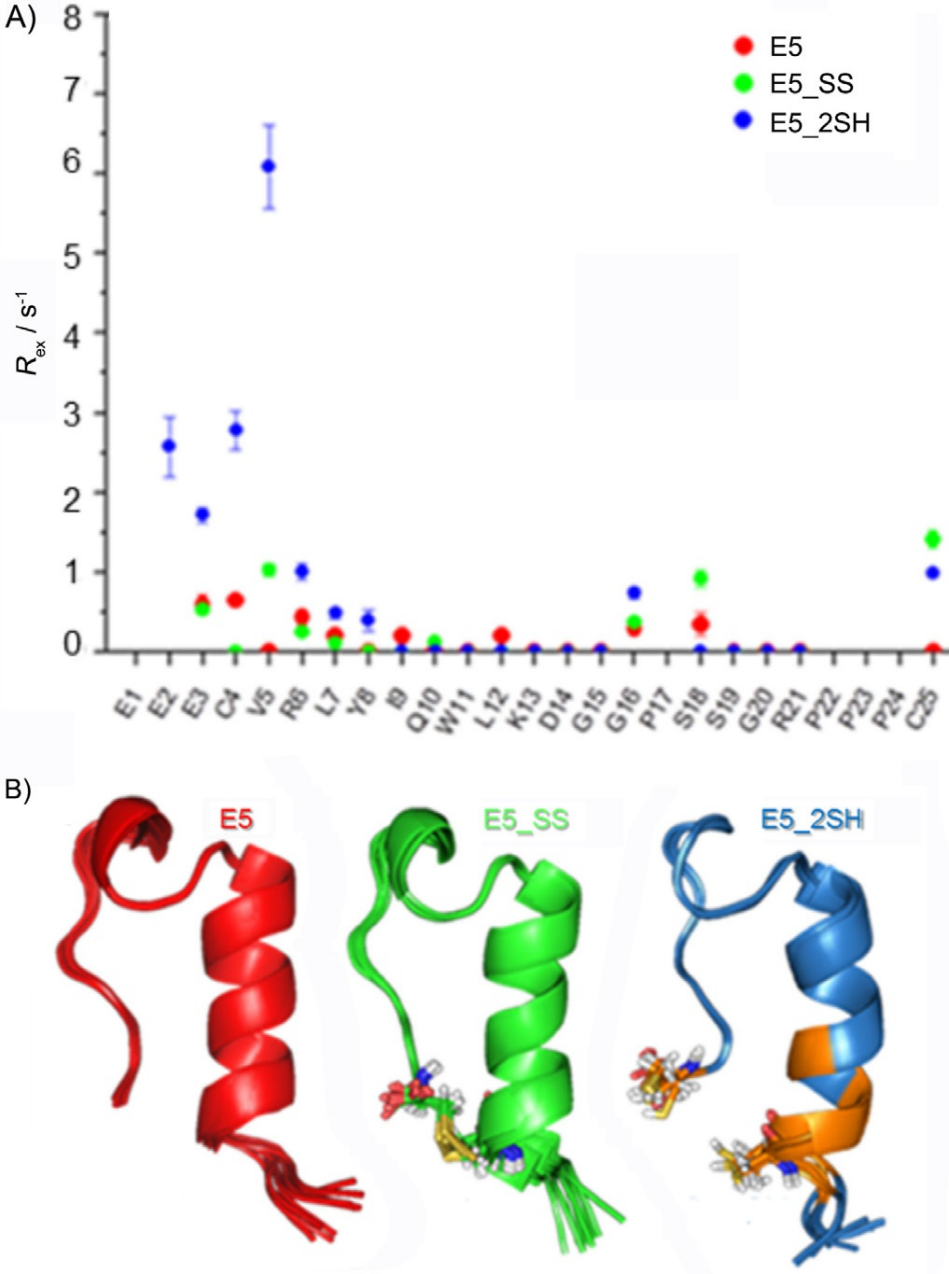
A) CPMG‐determined NH *R*_ex_ values of E5 (red), E5_SS (green), and E5_2SH (blue), and B) their backbone structures, with the key Cys residues highlighted. Slow exchange was measured for backbone NH groups of E3, C4, V5, R6, Y8, and C25 of E5_2SH only. Notably, residues that give *R*_ex_ are in the proximity of the Cys residues colored orange.

Capturing internal backbone dynamics occurring on the timescale of micro‐ to milliseconds was successfully attempted by means of Carr–Purcell–Meiboom–Gill (CPMG) NMR spectroscopy.^70^ Herein, we present the characterization of E5, E5_SS, and E5_2SH as examples. We found that only the backbone NH groups of Glu3, Cys4, Val5, Arg6, Tyr8, and Cys25 of E5_2SH partake in such slow motion. Considering the fact that all of these NH groups are close to both Cys residues (Figure 6), the CPMG data suggest that either E5_2SH presents alternative backbone structures, which interconvert at a slow exchange rate, or, due to incomplete reduction, the remaining oxidized form (1–8 %, see a discussion of the conversion rate below) constantly interconverts with the reduced form. The minor amount of coexisting oxidized form (E5_SS) could contribute to the stabilization of the dominant backbone fold of E5_2SH. The conformational equilibrium between the oxidized and reduced states seems to be the most likely explanation for the above‐described slow exchange; however, both scenarios of motion can occur in a concerted way.

### Modeling of the SS bond reduction kinetics

The SS bond reduction by TCEP is a bimolecular nucleophilic substitution (S_N_2) reaction.^37^ Thus, both the concentration of the oxidized form of the protein [_SS] and that of the RA contribute to the rate of the reduction. In an ideal case, we should consider only nucleophilic attack of the RA (*k*_1_), but, as we explained previously, in practice, we also have to take into account back oxidation (*k*_2_), which takes place simultaneously, and, in some cases, depending on the size and shape of the protein, precipitation (*k*_3_; Figure 5). The mechanism of reduction, therefore, can be described by Equations (2), (3), (4):

(2) $$\mathrm{SS}+\mathrm{RA}{}_{\mathrm{red}}\overset{k{}_{1}}{\underset{}{\to }}2\;\mathrm{SH}+\mathrm{RA}{}_{\mathrm{ox}}$$

(3) $$2\;\mathrm{SH}+O{}_{2}\overset{k{}_{2}}{\underset{}{\to }}\mathrm{SS}$$

(4) $$2\;\mathrm{SH}\overset{k{}_{3}}{\underset{}{\to }}\mathrm{SS}{}_{\mathrm{precipitated}}$$

By fitting this model to the concentration–time functions determined by means of NMR spectroscopy, *k*_1_, *k*_2_, and *k*_3_ can be determined, and half‐lives can be calculated. We focused on the determination of the reduction rate constants *k*_1_; therefore, sampling was more frequent in the reduction phase (stage I; Figure 5 B). Based on parameter estimation (see the Experimental Section), *k*_2_ and *k*_3_ are very often either negligibly small, or, due to a lack of sufficient data, cannot be confidently estimated. Obtaining key kinetic parameters allowed us to describe and compare the reduction kinetics of the SS‐containing miniproteins under various experimental conditions. Some protocols reported in the literature apply extreme conditions, such as high temperature (e.g., 50–80 °C), to obtain short reduction times; this is clearly unsuitable for maintaining the integrity of the protein, or >20‐fold molar excess of reagent. By performing the reduction of E19_SS (0.8 mm) under such conditions (60 °C with 18‐fold TCEP excess), the reaction seemed almost instantaneous (*t*_1/2_<5 min), but the sample became opalescent and side reactions (e.g., precipitation) were instantly detected. Similarly to most globular proteins, the conformational ensemble of E19_2SH at 60 °C is distinctly different from that of 15 °C; thus presenting many more unfolded states. The folded fraction of E19_2SH is 64 % at 15 °C, whereas it is 41 % at 60 °C, according to FUV‐ECD analysis. Instead of intramolecular reoxidation, undesirable intermolecular reoxidation might occur between particles. (Reducing E19_SS for 120 min, followed by centrifugation gave practically zero soluble protein concentration.) In general, reduction and reoxidation at higher *T* (e.g., ≥60 °C) is expected to be less effective, and accompanied by multiple side reactions, such as β‐elimination^71^ (which already occurs at a lower *T*),^72^, ^73^ racemization,^74^, ^75^ and aggregation. In principle, the reduction rate can be enhanced at lower *T* by increasing the TCEP molar ratio (15–20‐fold molar excess); however, this also triggers obscure unwanted processes (Figure S5). Experiments were repeated at different temperatures (15, 25, and 37 °C) with 0.8 mm protein and 18‐fold excess of TCEP (Table 2 and Figure S6). The Arrhenius equation allows the activation energy (*E*_a_) of the redox reaction to be derived, resulting in a value of about 44.3 kJ mol^−1^. For comparison, the activation energy of thiol–disulfide exchange between methylthiolate and oxidized DTT was calculated to be 62 kJ mol^−1^.^76^ Both FUV‐ECD and NMR spectroscopy derived structural information support the high conformational similarity between E19_SS and E19_2SH; therefore, *E*_a_ is likely to be used for the redox reaction, rather than for the conformational switch between the two conformational states (Table 1). Based on the NMR spectroscopy derived signal integral analysis, the reduction was almost complete (≈94 %) and no sign of precipitation was detected at any temperature. Additional experiments were performed to investigate the effect of the protein/RA ratio as a practical perspective (Figure S7). The above‐described NMR spectroscopy methodology provides high‐resolution information about the reduction mechanism, relative to that of the more rapid NUV‐ECD approach, and thus, details of the reduction of all four −SS− protein models were obtained through NMR spectroscopy.

**Table 2:** Kinetic parameters of temperature‐dependent E19_SS reduction with 0.8 mm protein and 18‐fold excess of TCEP. For detailed results of parameter estimation, see Figure S6.

*T*	Elapsed time	Conversion	*k*_1_	*t*_1/2_	Relative stan‐
[°C]	to steady	rate [%]	[L mmol^−1^ min^−1^]	[min]	dard deviation
	state [h]				of *k*_1_ [%]
15	≈15	92	3.05×10^−4^	181	3.27
25	≈6	94	7.68×10^−4^	72	9.78
37	≈4	94	1.15×10^−3^	48	1.68
60^[a]^	n.d.	n.d.	n.d.	n.d.	n.d.

[a] n.d.: not determined.

### Kinetics of SS bond reduction influenced by steric factors

**Figure 7:**
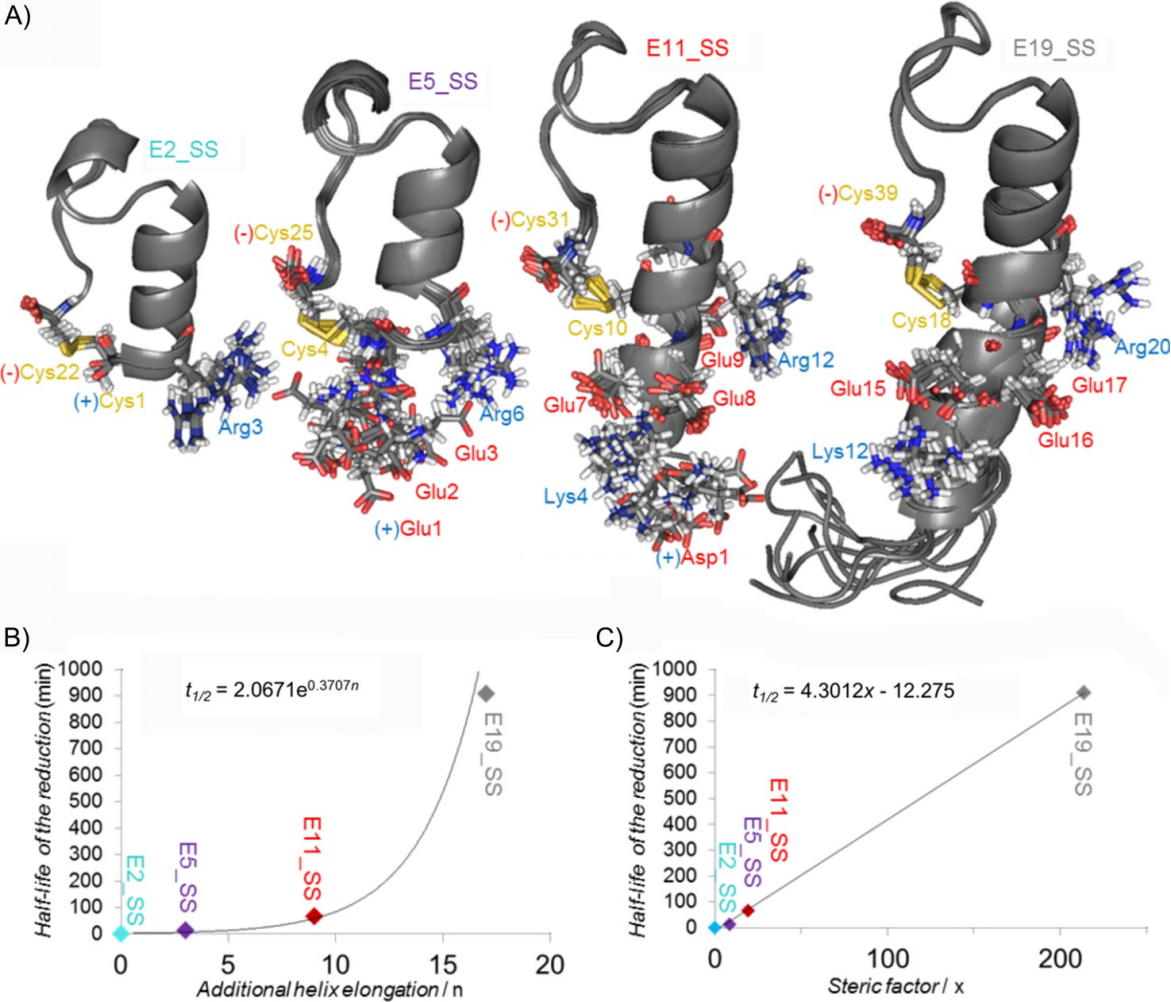
A) Ten superimposed structural ensembles of E19_SS, E11_SS, E5_SS, and E2_SS. Notably, all model proteins have their SS bonds at the surface, but their N termini are of different lengths and charges; thus, affecting the SS bond reducibility. Charged residues at pH 7, close to the reaction center are explicitly depicted: the negatively charged side chains are highlighted in red, whereas the positive ones are in blue. The C‐terminal negative charge of COO^−^ is marked by (−) and the amino group −NH_3_^+^ of the N terminus by (+). Correlation between the reduction half‐life versus B) helix length and C) steric factors (twofold excess of TCEP, 1.7 mm protein, 15 °C) is reported. (Figure S10 shows the correlation between the reduction half‐life and helix length in the case of DTT reduction.)

An appropriate reduction protocol was required to unambiguously determine the 3D structures of the above‐introduced pure reduced states. Thus, in agreement with the above discussion, only mild conditions (15 °C and twofold molar excess of TCEP) were used for the reduction of the four different miniproteins. Determining the structural properties and reduction rates under the same conditions allowed us to elucidate the basis of the observed differences in the reduction rates. We found that, at *T=*15 °C, the *k*_1_ values of these four model proteins, comprising of identical core structures, but different lengths, were indeed different: their *k*_1_ and *t*_1/2_ values strongly depended on their sizes and/or molecular weights. It appears as if “cutting back” on the α‐helical segment strongly affects the SS bond reducibility, even though the SS bonds of all four models are near the surfaces (Figure 7 A). To our great surprise, we recorded three orders of magnitude differences between the reduction rate constants (Table 3). Whereas the reduction of E2_SS is still extremely fast, tE2_SS1/2 <≈1 min, that of E5_SS occurs on the timescale of minutes: tE5_SS1/2 ≈14 min. E11_SS, which is elongated by six residues (1.5 turns of α‐helix) with respect to that of E5_SS, exhibits about a fourfold increase in *t*_1/2_ (tE5_SS1/2 ≈14 min→tE11_SS1/2 ≈67 min). Finally, the unstructured short octapeptide tail HAEGTFTS‐ further lengthens *t*_1/2_ by about 13‐fold (tE11_SS1/2 ≈67 min→tE19_SS1/2 ≈909 min). The conversion rate was close to complete for the shorter peptide of E2_SS, whereas the reduction of E19_SS was only 88 % complete. The kinetic parameters of all four model proteins were determined by using a twofold molar excess of DTT, at pH 7, and *T=*15 °C. The mechanism of SS bond reduction by DTT is also S_N_2,^77^ but the determined *t*_1/2_ values are significantly longer than those obtained by using the same molar excess of TCEP; however, the observed overall tendency and conclusion appear to be the same (Table 3).

**Table 3:** Kinetic parameters of the SS bond reduction of the four model proteins. For each reduction, a protein concentration of 1.7 mm was used with twofold excess of TCEP and DTT at 15 °C. For detailed results of parameter estimation, see Figures S8–S9. NMR spectroscopy derived structural properties of the outer helix are also shown.

TCEP						DTT					Properties of outer helix			
	Elapsed	Conversion	*k*_1_	*t*_1/2_	Relative	Elapsed	Conversion	*k*_1_	*t*_1/2_	Relative	Outer	RMSD	[∑CSDHα(i) ]	Steric
	time to steady state	rate [%]	[L mmol^−1^ min^−1^]	[min]	standard deviation of *k*_1_ [%]	time to steady state [h]	rate [%]	[L mmol^−1^ min^−1^]	[min]	standard deviation of *k*_1_ [%]	helix length (*i*)	of outer helix	/*i*	factor^[a]^
E19_SS	≈76 h	87	2.71×10^−4^	909	7.30	n.d.	n.d.	n.d.	30 545^[b]^	n.d.	17	1.41	0.11	213.74
E11_SS	≈5–6 h	94	3.68×10^−3^	67	2.61	138	≈84	1.52×10^−4^	1659	45.152	9	0.55	0.26	19.26
E5_SS	≈1 h	93	1.85×10^−2^	14	3.35	9–10	95	2.18×10^−3^	115	1.064	3	0.39	0.14	8.56
E2_SS	<5 min	100	2.59×10^‐−1^	≈1	15.37	5	100	4.04×10^−3^	62	5.315	0	0	0	0

[a] Steric factor comprises the following factors: the length of the outer helix, the RMSD, and the reciprocal value of [∑CSDHα(i) ]/*i*. [b] Half‐life of E19_SS reduction by DTT was calculated according to the equation of the dependence of the half‐life on outer helical length.

**Scheme 1:**
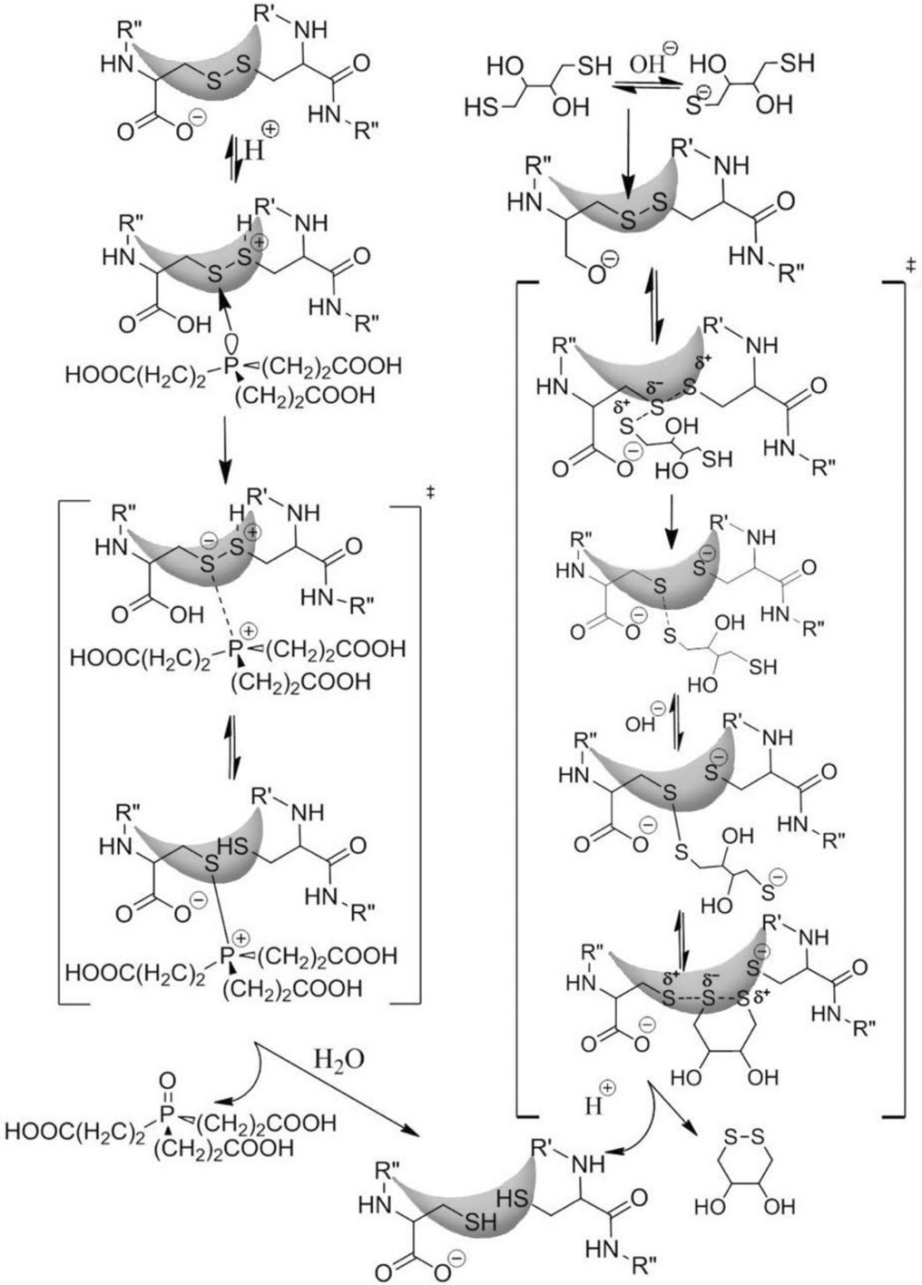
A generalized mechanism of TCEP‐ and DTT‐assisted mechanisms of SS bond reduction in proteins. Functional group R− stands for the N terminus of the protein systematically elongated here: in E19_SS the R group is equal to H^+^‐HGEGTFTSDLSKQMEEE‐, in E11: H^+^‐DLSKQMEEE‐, in E5_SS: R=H^+^‐EEE‐, and in E2_SS it is simply H^+^. A brief description of the detailed reaction mechanism is provided for both TCEP and DTT in the text.

Because the well‐folded Trp cage motifs are identical (based on their CSD cage values; Table 1) in all four model proteins, the observed *k*_1_ differences must be associated with the structural properties of their α‐helices and the eventually appearing unstructured tail. Although the dataset is limited (*n=*3 or 4), as the simplest approach, the length of the α‐helix (*n*) and the half‐lives (*t*_1/2_) of reduction could be correlated, leading to an exponential dependence for both TCEP (*t*_1/2_=2.06e^0, 371*n*^, *R* ^2^=0.95) and DTT (*t*_1/2_=50.47e^0, 377*n*^, *R* ^2^=0.98) as the RA (Figure 7 B). To take into account the additional structural descriptors for a more complete characterization, we derived the steric factor (*x*) for these protein models [Eq. (5)]:

(5) x=1[∑CSDHα(i)]/i×RMSD×n

in which the reciprocal of the helicity ([∑CSDHα(i) ]/*i*) and the bulkiness (RMSD) of the outer helical part were both calculated with respect to the length of the N terminus (*n*; Table 3). We observed a linear dependence of the steric factors on the reduction half‐lives as a function of the length of the N terminus (Figure 7 C). Some, but not all, of the above *k*_1_ (*t*_1/2_) differences can be explained by structural differences of the outer helix because both solvent exposure and local charges around the SS bonds are also different. In Scheme 1, we provide a summary of the mechanistic explanation, including all of these factors and viewpoints.

### Rate‐determining steric and electronic factors of SS bond reduction

Apart from the steric effect of the helical part emphasized above, the S_N_2 mechanism of TCEP‐driven reduction has to be discussed in terms of electrostatic effects.^19^ In general, attack is more favorable and effective on those structures in which the C terminus is neutral. According to the average p*K*_a_ of the cysteine carboxyl group (p*K*_a_=1.92) at pH 7, the proportion of COOH/COO^−^ is low: 1/12 000. The rate‐determining step is cleavage of the SS bond.^78^ During the S_N_2 reaction, the nucleophilic P atom of TCEP attacks one of the SS bonds, forming a thiophosphomium salt (an S^−^−P^+^ ion‐pair complex; Scheme 1).

Nucleophilic attack (n→σ*) is facilitated by the favorable arrow‐shaped (tetrahedral: 105°) steric arrangement of the nonbonding electron pair of the P atom of TCEP. The main portion of the activation Gibbs free energy of reduction is consumed by splitting of the SS bond and not by the steric rearrangement of the intermediate structure.^79^ Better solvation of the thiol and zwitterion results in a lower activation Gibbs free energy of the reaction. Next, the positively charged −S−P^+^−[(CH_2_)−COOH]_3_ complex hydrolyzes rapidly and results in the phosphine oxide and free −SH groups of the protein.

Both charged and aromatic side chains can participate, and thus, intimately influence the efficacy of TCEP‐mediated reduction (Figure 7 A). The nucleophilic phosphine attacks the C‐proximal cysteine because the intermediate cation can be stabilized by the proximal COO^−^ group of the C‐terminal cysteine. A positive charge near the SS bond could enhance the reaction through electrostatic compensation of the N‐proximal leaving thiolate group, whereas a negative charge might slow down the S_N_2 reaction.^80^, ^81^ Direct through‐bond effects of any charged side chain can be ignored because they are separated by several σ bonds from the negative COO^−^ group. Although the inductive or direct σ‐bond effects are negligible, both steric and spatial electrostatic effects in the vicinity of *N*‐proximal cysteine play a major role in the reduction rate. At pH 7, the positively charged Arg near the SS bond in the inner helix may facilitate reduction; however, it is distant from the SS bond (Figure 7 A), and thus, a direct charge‐controlled interaction is less likely to occur. On the other hand, the positively charged N‐terminal −NH_3_^+^ can directly catalyze the instantaneous reduction^82^ of E2_SS (tE2_SS1/2 ≈1 min) because H−N−C^α^−C^β^−S of the cysteine forms a five‐membered pseudo‐ring that facilitates intramolecular NS proton transfer.^83^ Thus, upon TCEP attack, these ideal local electrostatic compensations may stabilize the intermediate thiophosphonium salt, shifting the reaction equilibrium towards splitting of the SS bond. Furthermore, because the leaving thiolate anion is only positioned at the N terminus of the well‐folded α‐helix, the positive charge of the α‐helix macrodipole also promotes progress to the reduced state.^84^, ^85^ Moreover, due to the small protein size, the SS bond is most exposed to solvent and reagent in E2_SS.

As the N terminus is elongated on the α‐helix from E2_SS toward E19_SS, the “catalyzing” −NH_3_^+^ group of the N terminus moves further away from the SS bond, and the effect of the macrodipole gradually vanishes; thus, the reduction rate is reduced (*t*_1/2_ increases; Table 3). The role of this positive charge was directly probed by acetylating the N terminus, Ac‐E2_SS, and, as expected, the half‐life of reduction increased significantly: tE2_SS1/2 ≈1 min→tAc-E2_SS1/2 ≈8 min (in both cases, a protein concentration of 1.7 mm and twofold excess of TCEP were used).

The N‐terminal elongation of E2_SS by three Glu residues results in E5_SS. As expected, the reduction rate is slower: tE5_SS1/2 ≈14 min. Although only a tripeptide is added to the dynamic N terminus, reaching the SS bond still becomes harder for both reagent and/or solvent molecules. In addition, the 3D structure (Figure 7 A) shows that the three negatively charged Glu side chains (at pH 7) are flanked by the N‐proximal cysteine and the positively charged N terminus, and thus, effectively neutralize the catalytic effect. The structure of the ensemble determined by means of NMR spectroscopy shows a distance fluctuation from 3.7 to 10.7 Å between 4Cys Cβ and 1Glu NH_3_^+^, whereas that of 4Cys Cβ and 1Glu COO^−^ fluctuates between 3.4 and 12.4 Å (Figure 7). Thus, SS bond protonation requires an active contribution from the medium; but proton transfer is perturbed by the proximity of the negatively charged glutamate side chains.

Further elongation of E5_SS by the hexapeptide ‐DLSKQM‐ leads to E11_SS. Under the same conditions, the reduction of this even larger model protein occurs more slowly (tE11_SS1/2 ≈67 min). The glutamate side chains are more oriented by the longer α‐helix of E11_SS (Figure 7): whereas 8Glu^−^ turns outward, both 7Glu^−^ and 9Glu^−^ flank the SS bond from two sides. Residues 7Glu^−^ with 4Lys^+^ and 9Glu^−^ with 12Arg^+^ are capable of forming salt bridges in close vicinity, and thus, could partly compensate for the slowing effect of the negatively charged side chains. E11 was found to be more helical than that of longer E19;^56^ thus we find here that both E11_SS and E11_2SH have more compact α‐helices than those of E19_SS and E19_2SH, according to both [∑CSDHα(i) ]/*i* NMR spectroscopy measurements and FUV‐ECD spectral properties. We believe that, in addition to partly compensated for negative electrostatic effect(s), mainly steric effects of the elongated and stiffer α‐helix cause the longer value of tE11_SS1/2 with respect to that of tE5_SS1/2 .

Finally, E11_SS elongated by the ‐HGEGTFTS‐ octapeptide results in E19_SS—the largest model protein used herein—for which the longest half‐life (tE19_SS1/2 =909 min) is measured. E19_SS has the same electrostatic pattern in the vicinity of the SS bond as that of E11_SS, but its reduction rate is about 15 times slower than that of E11_SS. Although the ‐HGEGTFTS‐ segment is far from the SS bond (*d*_7Thr‐18Cys_=11–14 Å; Figure 7) and cannot influence reduction by electrostatic interactions, its higher internal dynamics (low *S* ^2^ value),^56^ as a steric effect, must slow the SS bond reduction rate further. In fact, the latter increase, in terms of *t*_1/2_, is a good estimation of the magnitude of a purely steric effect of an unstructured polypeptide chain on reduction rate.

### Differences in reduction kinetics and mechanism with alternative reagents

There are a few distinct differences in terms of the general mechanism of SS reduction by TCEP and DTT (Scheme 1). 1) As an initializing step, deprotonation of the thiol group of DTT is required for successful nucleophilic attack, which depends on the pH of the medium. According to the Henderson–Hasselbach equation,^86^ taking into account the acidic dissociation constant of DTT (p*K*_a1_=9.2 and p*K*_a2_=10.1) at pH 7, deprotonated thiolate concentration is about three to four times lower than that of the overall DTT concentration. After successful nucleophilic attack on the SS bond, a linear −S−S−S− transition complex has to be formed, in which the negative charge is located on the two leaving S atoms.^87^ An intramolecular protonation, as for TCEP, also stabilizes the thiol anion leaving group if DTT is used, and thus, enhances the reaction rate. Therefore, a positive inductive/steric effect increases, whereas a negative effect decreases the reduction rate. 2) Contrary to TCEP, the active species of DTT has a negative charge. Therefore, charged amino acid side chains close to the SS bond will directly affect attack by the nucleophilic RA. In line with these observations, both the negative C terminus and the SS bond flanking glutamate side chains repel DTT; thus contributing to a significant and large‐scale decrease in reaction rate (Table 3). 3) Moreover, complete reduction by DTT consists of two steps: after the first attack, the free SH group of the peptide–DTT complex has to cleave the previously formed SS bond, whereas DTT closes into a six‐membered ring (Scheme 1). All of these factors jointly decrease the reduction rate if DTT is used instead of TCEP (Table 3). These considerations make it even more striking that, although several proteins with various numbers of SS bonds per molecule, such as α‐lactalbumin, lysozyme, and oxytocin, were reported to be completely reduced in 5 min by 10 mm DTT at pH 5.5 and 70 °C,^88^ we found that the reduction of miniproteins (e.g., E11_SS) with a single and exposed SS bond might take up to 138 h (Table 3).

### Spontaneous SH reoxidation accompanied by polymerization

**Figure 8:**
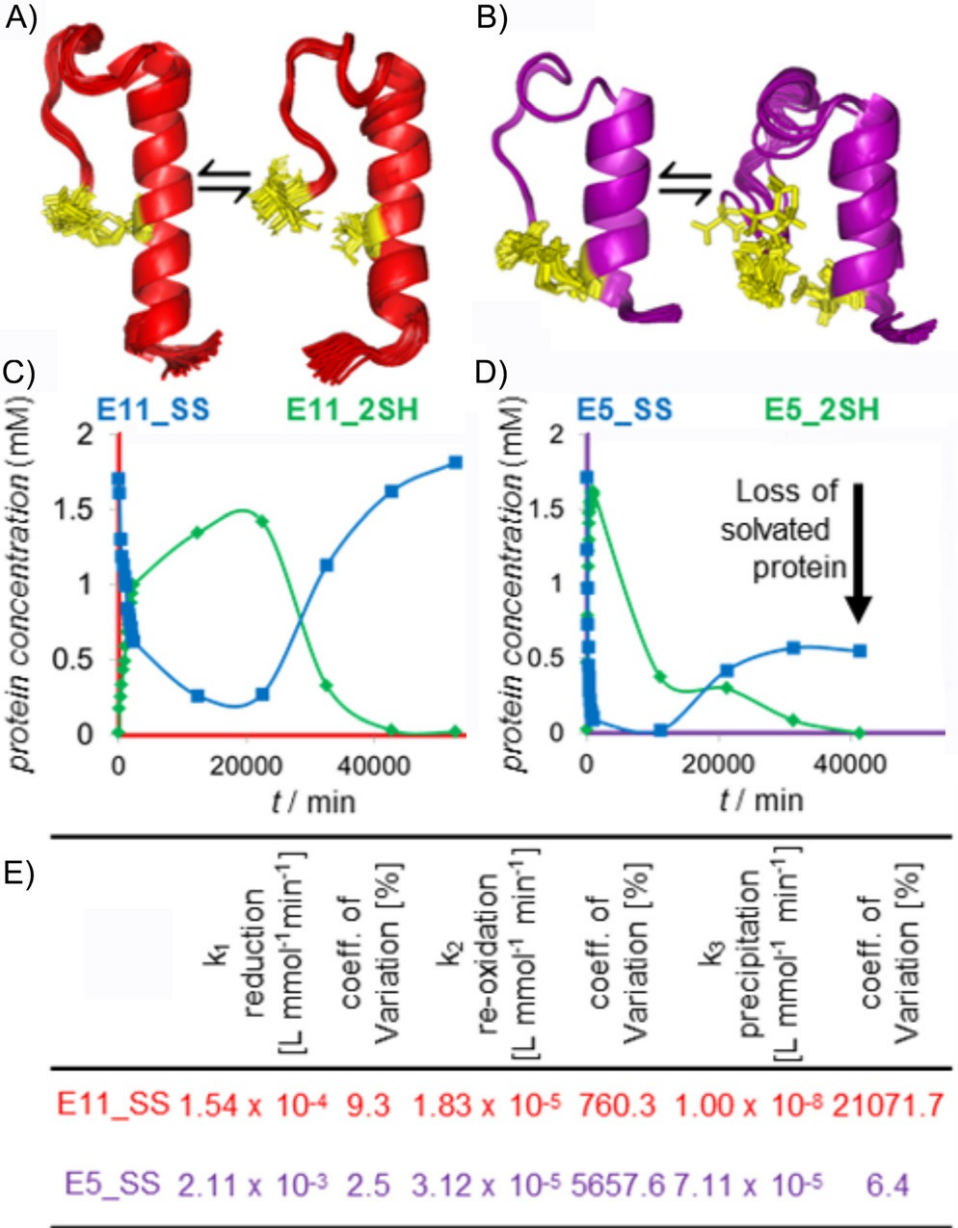
The 50‐membered structure ensembles of A) E11_SS⇄E11_2SH and B) E5_SS⇄E5_2SH. The fold of E11_2SH is more compact than that of E5_2SH, which has more “open” conformers, in which the Cys residues are far from each other. This allows intermolecular, rather than intramolecular, reoxidation. The dissolved oxidized and reduced protein concentrations of C) E11_SS⇄E11_2 SH and D) E5_SS⇄E5_2SH (oxidized: blue; reduced: green) as a function of time. In the case of E5_SS⇄E5_2SH, the initial concentration decreased by 68 %, whereas, at the end of a complete redox cycle, the concentration of E11_SS⇄E11_2SH remained the same. E) Estimated parameters of the complete redox cycles. (The *k*_1_ values are slightly different from those in Table 3, for which the estimation comprises data only for phase 1.) Notably, in these long‐term experiments, the rate of O_2_ diffusion characterized by the rate constant *k*_4_ was also involved. Figure S11 contains all data for parameter estimation of E11_SS, E5_SS, and E2_SS.

Incomplete conversion, despite the presence of a large excess of the RA, provided evidence for the reoxidation of the reduced SS bond of the studied model systems. To study this process in detail, the in situ reoxidation of the DTT‐reduced protein samples at room temperature in sealed NMR tubes (pH 7, 15 °C, twofold excess of DTT) was monitored for several weeks. Spontaneous reoxidation of E2_2SH, E5_2SH, and E11_2SH by dissolved O_2_ was clear after four weeks (Figure 8). The reoxidation rates (*k*_2_) have comparable orders of magnitude to that of the reduction rates (lower by one order of magnitude), but reoxidation has a pronounced role only after reaching steady state, at which the concentration of the already reduced peptides becomes significant.

Reoxidation can take place both intra‐ and intermolecularly. Whereas the former leads to a decrease of overall conversion rates, the latter results in the formation of random molecular clusters, which may lead to precipitation. According to our semiquantitative analysis based on the recorded ^1^H NMR spectra, the integral changes of the Trp H*ϵ*1 resonances both in the oxidized and reduced forms of the protein during reduction with DTT show a decrease in concentration over the observed period of redox time for both E2_SS and E5_SS. Precipitation can be more intense if the protein concentration is higher. According to our present observations, increasing the length of the α‐helix within the Trp cage proteins stabilizes the soluble protein fraction. This means that the elongated N terminus, namely, the outer helix in the case of E11_2SH, effectively shields the free SH− groups of the reduced protein, and thus, prevents any intermolecular reoxidation, whereas shorter variants, such as E2_2SH and E5_2SH, yield a significant amount of polymer formation. Due to the diversity of open 3D folds of both E5_2SH and E2_2SH, spontaneous intramolecular ring closure is hindered and less likely to happen. The N‐terminal Cys of E11_2SH is placed and fixed at the highly ordered inner helix, with a reduced internal mobility of Cys18, and thus, mostly intramolecular ring closures take place. In the case of E5_2SH, intermolecular SS bond formation is allowed, but may be limited just by Brownian motion and concentration. A comparison of the polymerization rates (kE11_SS3 <kE5_SS3 ) with different N‐terminal lengths also supports this concept (Figure 8 E).

E2_SS was N‐acetylated to eliminate the reduction rate‐enhancing effect of the positively charged N terminus, −NH_3_^+^, in the vicinity of the SS bond. Upon acetylation, *t*_1/2_ has indeed increased (tE2_SS1/2 =≈1 min tAc-E2_SS1/2 =≈8 min), but, in addition, the reaction reaches its steady state at a low conversion rate (50 %). During reduction, almost immediately, both of the appropriate signal integrals of Ac‐E2_SS and Ac‐E2_2SH start to decrease, with a foamy precipitate gradually forming in the NMR tube. The isolated and HPLC‐purified precipitant was identified as a polymer of the parent miniprotein by means of MS (Figure S12). Oligomer formation and soluble protein concentration decrease were more advanced for Ac‐E2_SS than that of E2_SS (Figure S13). Due to the absence of the shielding effect of the outer α‐helix, the free thiol moiety of the N terminus is accessible for additionally reduced peptides in which the two free SH groups can hook peptide chains together. The polymer can grow until another free N terminus and acetylated C‐terminal thiol‐containing peptide closes polymerization. In addition, for Ac‐E2_2SH, intramolecular N→S acyl transfer could take place,^89^ blocking some of the SH groups from promoting oligo‐ and polymerization through intermolecular SS bond formation.

## Conclusion

The SS‐bond cyclized exenatide derivate and its variants were synthesized. Both the oxidized (E19_SS) and reduced (E19_2SH) forms, along with the parent molecule, E19, and all three of their truncated variants (E11_SS, E11_2SH, E11, E5_SS, E5_2SH, E5, E2_SS, E2_2SH, and E2) comprised the same Trp cage/SS/SH bond motif as that of their core structures. The SS bond stabilized model proteins showed improved thermostability and 3D fold compactness, with respect to their reduced and parent forms. Key residues for receptor binding remained in position in all of these models; therefore, E19_SS might be promising agonists for GLP‐1R and as a lead compound for type 2 diabetes mellitus.

The reduction rate of E19_SS was found to be unexpectedly slow compared with that reported in the literature. The reaction takes hours (*t*_1/2_=48 min), even at 37 °C, although the protein is small, and its single SS bond is exposed at the surface, and thus, accessible for reducing reagents. All four Trp cage variants studied herein have an almost equally compact core structure, with α‐helical segments of different length and internal mobility. By performing a complete NMR spectroscopy based structure elucidation, we found that the progress of reduction could be monitored by means of ^1^H NMR by using selected resonance frequencies. We have established that these four model proteins of different α‐helical lengths have significantly different reduction rate constants. Although it is generally complicated to discriminate each factor that affects the SS bond reduction rate, the present set of miniproteins enabled them to be deciphered separately. We have focused special attention on the importance of the intramolecular protonation of the SS bond; this step greatly enhances the reaction rate. From CPMG measurements, we found that, at steady state, selected residues in the vicinity of the SS bond presented a slow exchange on the micro‐ to millisecond timescale of motion. This redox cycle lasts as long as active RA can be found in solution. We found that structural, steric, and electrostatic factors influenced the reduction rate greatly, resulting in almost three orders of magnitude differences in reduction half‐lives (*t*_1/2_) for otherwise structurally similar and globularly folded model proteins.

Notably, in addition to intramolecular reoxidation within the redox cycle, intermolecular oxidation could also occur. The rate of these two concerted reactions depended on 1) the internal dynamics of the backbone conformers in the proximity of the SS bond, and 2) the shielding effect of the α‐helix on the SS bond. Intramolecular N→S acyl transfer in Ac‐E2_SS inhibits intramolecular reoxidation, but increases intermolecular reoxidation, which leads to oligo‐ and polymerization.

We found that easy‐to‐collect NUV‐ECD spectral properties were indeed useful for monitoring the SS→SH reaction, even quantitatively, without the time‐consuming assignment of the high‐resolution NMR spectroscopy data. If the SS bond were situated in the vicinity of an aromatic cluster, NUV‐ECD spectral changes could be used to monitor the transformation, which was proportional to the extent of reduction and clearly signaled when steady state had been reached. Thus, we encourage the use of CD spectroscopy for monitoring protein reduction rate in the manufacture of recombinant proteins (e.g., insulin, human monoclonal IgG antibodies) on a large scale, to control and provide information on the state of SS–SH bonds.

## Experimental Section

**ECD**: FUV‐ECD spectra were recorded on a Jasco J810 spectrophotometer by using a 1.0 mm path length cuvette with protein concentrations of 20–30 μm. Data accumulation was performed over a range of 185–260 nm, with 0.2 nm step resolution at a scan rate of 50 nm min^−1^ with a 1 nm bandwidth. The spectral accumulations were resolved between 5 and 85 °C in steps of 5 °C. The temperature was controlled by using a Peltier‐type heating system. Each spectrum baseline was processed by subtracting the solvent spectrum from that of the protein and the raw ellipticity data were converted into mean residue molar ellipticity units, [*Θ*]_MR_.

**Reduction monitoring by NUV‐ECD**: The spectra were recorded on a Jasco J810 spectrophotometer by using a 10 mm path length cuvette with protein concentrations of 120–150 μm. Data accumulation was performed over a range of 240–325 nm, with 0.2 nm step resolution at a scan rate 50 nm min^−1^ with a 1 nm bandwidth. The sample was tempered by using a Peltier‐type heating system. Each spectrum baseline was processed by subtracting the solvent spectrum from the peptide spectrum and the raw ellipticity data were normalized by the concentration [*Θ*]. Reduction was followed for 75 h. Each intensity [*Θ*] at 266, 281, 287, and 293 nm was converted into concentration by using Equation (6).

(6) $$c\left(t\right)=\frac{A{}_{\infty }-A}{A{}_{\infty }-A{}_{0}}\left[\mathrm{SS}\right]{}_{0}$$

**NMR spectroscopy**: All ^1^H NMR spectroscopy experiments were performed on a Bruker Avance III 700 MHz spectrometer equipped with a *z*‐gradient 5 mm probe head operating at 700.13 MHz for ^1^H, whereas ^31^P NMR spectroscopy experiments were performed on a Bruker Avance 250 spectrometer with a 5 mm SB quad probe head.

**Monitoring reduction kinetics**: Peptide samples were prepared between 0.8 and 1.8 mm in 50 mm NaH_2_PO_4_–Na_2_HPO_4_ buffer (600 μL, pH 6.95), with 10 % D_2_O. A 0.1 m solution of NaOH was used to set the pH to 7. Sodium trimethylsilylpropanesulfonate (DSS) was added as the internal proton reference standard, set to *δ*=0.0 ppm under all conditions. ^1^H,^1^H 2D homonuclear spectra were recorded for the oxidized peptide; thereafter, upon the addition of a different excess of 0.5 m TCEP or DTT, reduction was observed by recording a series of 1D ^1^H spectra (ns=64 or 128 scans). Finally, at the end point, ^1^H,^1^H homonuclear 2D spectra were recorded on the reduced peptide. Data sets were processed by using TopSpin 3.2 software. The conversion rate was determined by using the relative integral of the Trp H*ϵ*1 signal in the oxidized (Int_OX_) and reduced (Int_RED_) form. Each integral was normalized to the integral of DSS. The concentrations were determined by the ratio of the oxidized and reduced integrals and the initial protein concentration.

**Structure determination**: ^1^H NMR spectroscopy assignations were completed by using ^1^H,^1^H COSY and ^1^H,^1^H TOCSY spectra, and then the distance restraints were determined based on ^1^H,^1^H NOESY spectra. Spin locks for ^1^H,^1^H TOCSY were 80 ms, whereas the mixing time for ^1^H,^1^H NOESY was 150 ms. CCP NMR^90^ spectroscopy was used for resonance assignment, crosspeak calibration, and structure refinement. CNS Solve 1.3,^91^ Aria 2.0 standard iteration protocol, and water refinement were used for 10‐membered structure ensemble calculations. All structural figures were illustrated by using PyMOL software.

**CPMG effect**: Backbone ^15^N‐longitudinal (*R*_1_) and transverse (*R*_2_) relaxation rates and the heteronuclear ^1^H,^15^N cross‐relaxation rate constant (NOE) of E5, E5_SS, and E5_2SH were measured at 288 K. For each crosspeak (*i*), *R*_2,i_ values were calculated by using Equation (7):

(7) $$R{}_{2,i}=\frac{-\ln\left(\frac{I{}_{i}}{I{}_{\mathrm{ref}}}\right)}{\frac{t{}_{\mathrm{CPMG}}}{s}}$$

in which $I_{i}$ is the intensity of the given crosspeak in the *i* th spectrum, *I*_ref_ is the intensity of the given crosspeak in the reference spectrum, and *t*_CPMG_ is the relaxation period of the CPMG measurement. The *R*_2_ values per residues were plotted against *ν*_CPMG_ [Hz]. Quantitative analysis of the CPMG graph reveals those residues that show CPMG effects in the protein.

**Peptide synthesis and purification**: Proteins were prepared by means of standard solid‐phase peptide synthesis or bacterial expression methods, as published previously.^92^ Proteins were purified by means of reversed‐phase HPLC on a C_18_ column by using a gradient of water/acetonitrile eluents. (Eluent A: 0.1 % trifluoroacetic acid (TFA) in water; eluent B: 0.08 % TFA and 80 % acetonitrile in water.)

**Parameter estimation**: Kinetic parameter estimation was based on the integral of selected NMR signals considered to be proportional to the concentration of the relevant species. The mechanism taken into account is that given by Equations (8), (9), (10), (11):

(8) $$\mathrm{SS}+\mathrm{Red}\overset{k{}_{1}}{\underset{}{\to }}2\;\mathrm{SH}+\mathrm{Ox}$$

(9) $$2\;\mathrm{SH}+O{}_{2}\overset{k{}_{2}}{\underset{}{\to }}\mathrm{SS}$$

(10)
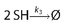

(11)
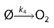



in which SS is the reduced model protein with an intramolecular S−S bond; 2 SH is the same protein with the S−S bond reduced to two −SH groups; and the symbol Ø means a different phase from that of the reaction mixture, that is, the polymer aggregate as a sink in the first case and the gas phase as a source in the second case. Notably, in some cases, in which polymer precipitation (*k*_3_) and/or oxygen diffusion (*k*_4_) from the gas phase proved not to be present (indicated by largely nonsignificant estimated parameters concerning these processes), these steps have been omitted from the fitted mechanism.

For parameter estimation, the COPASI 4.16 (Build 104) Biochemical System Simulator software (http://copasi.org/) was used, with the parameter estimation option of the Levenberg–Marquardt method. The result of the estimation procedure did not depend on the choice of the initial parameters within a large interval; thus, there was one stable optimum for the fit of the model only. Confidence interval half‐widths and relative standard deviations based on them were calculated from the estimated standard deviations, which suggested a Student distribution with *n*−*p* degrees of freedom, in which *n* is the number of data points in the concentration versus time measurements and *p* is the number of parameters estimated.

To determine the half‐life and initial concentration of the SS species, a kinetic analysis of the temporal evolution of the reactions was performed. Both reduction and oxidation proved to be second‐order reactions, which was not only supported by the good fit of the model, but also by the fact that, with this mechanism, the measured ([SS]_0,meas_) and calculated ([SS]_0,calcd_) initial concentrations of the model proteins were in very good agreement. From the kinetic analysis, the initial concentration of oxygen ([O_2_]_0_) could also be estimated, except for one case in which the uncertainty of this parameter was very large, due to the lack of sufficient experimental data.

Because reduction follows second‐order kinetics, the half‐life (*t*_1/2_) of model proteins also depends on the actual concentration of the RA in the reaction mixture [Eq. (12):

(12) $$t{}_{1/2}=\frac{1}{k{}_{1}\left(c{}_{\mathrm{Red},0}-c{}_{\mathrm{SS},0}\right)}\ln\left(2-\frac{c{}_{\mathrm{SS},0}}{c{}_{\mathrm{Red},0}}\right)$$

in which *k*_1_ is the rate constant of reduction, *c*_SS,0_ is the initial concentration of the model protein, and *c*_Red,0_ is the initial concentration of the RA. [Notably, Eq. (12) is valid only if *c*_Red,0_ is greater than *c*_SS,0_—as in the current case. If *c*_SS,0_ exceeds *c*_Red,0_, but it is not higher than that of twice the value of *c*_Red,0_, then the two initial concentrations should be flipped in both the difference and the fraction. If *c*_SS,0_ exceeds *c*_Red,0_ by more than a factor of two, then the SS protein concentration cannot become as low as half of the initial concentration, due to reduction.] For this reason, the half‐life is less indicative of the rate of hydrolysis; the correct comparison of the rates can be made based on the rate constant(s) of the second‐order reaction(s).

## Conflict of interest

*The authors declare no conflict of interest*.

## Supporting information

As a service to our authors and readers, this journal provides supporting information supplied by the authors. Such materials are peer reviewed and may be re‐organized for online delivery, but are not copy‐edited or typeset. Technical support issues arising from supporting information (other than missing files) should be addressed to the authors.

Supplementary
