# Supplementary material for: Compactness of Protein Folds Alters Disulfide‐Bond Reducibility by Three Orders of Magnitude: A Comprehensive Kinetic Case Study on the Reduction of Differently Sized Tryptophan Cage Model Proteins
Source: Chembiochem. 2019 Nov 18;21(5):681–95. doi: 10.1002/cbic.201900470 (PMC7079008; doi:10.1002/cbic.201900470)
Supplement: Supplementary file 1 — Supplementary [file CBIC-21-681-s001.pdf]

CHEMBIOCHEM

Supporting Information

Compactness of Protein Folds Alters Disulfide-Bond Reducibility by Three Orders of Magnitude: A Comprehensive Kinetic Case Study on the Reduction of Differently Sized Tryptophan Cage Model Proteins

Dániel Horváth,[a] Nóra Taricska,[a] Ernő Keszei,[b] Pál Stráner,[a] Viktor Farkas,[a] Gábor K. Tóth,[c] and András Perczel*[a]

cbic_201900470_sm_miscellaneous_information.pdf

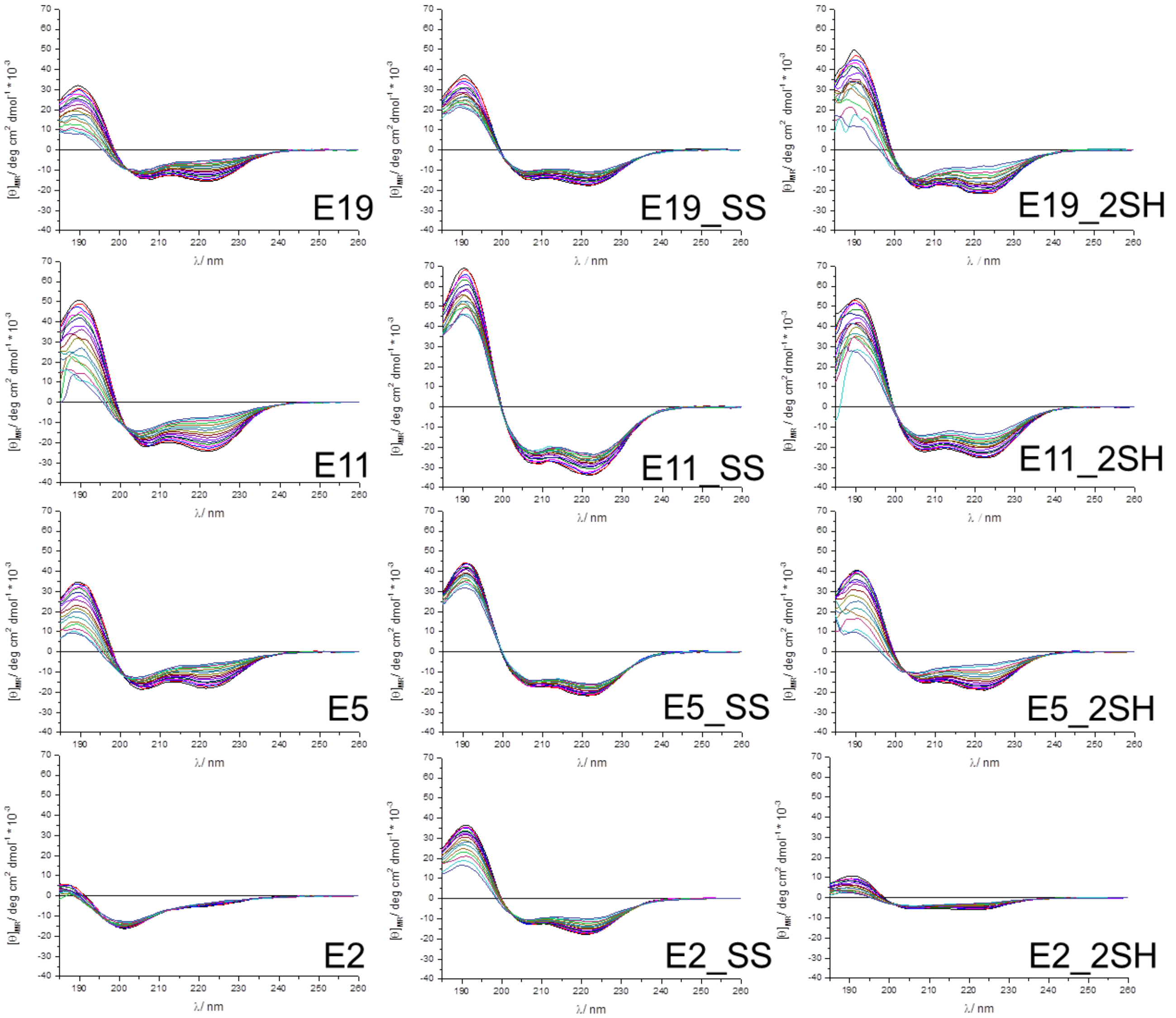

Supplementary Figure 1. Temperature dependent (5–85 °C) far-UV ECD spectra (185–260 nm) of parent, SS-bond enhanced and SS-bond reduced Exenatide derivates.

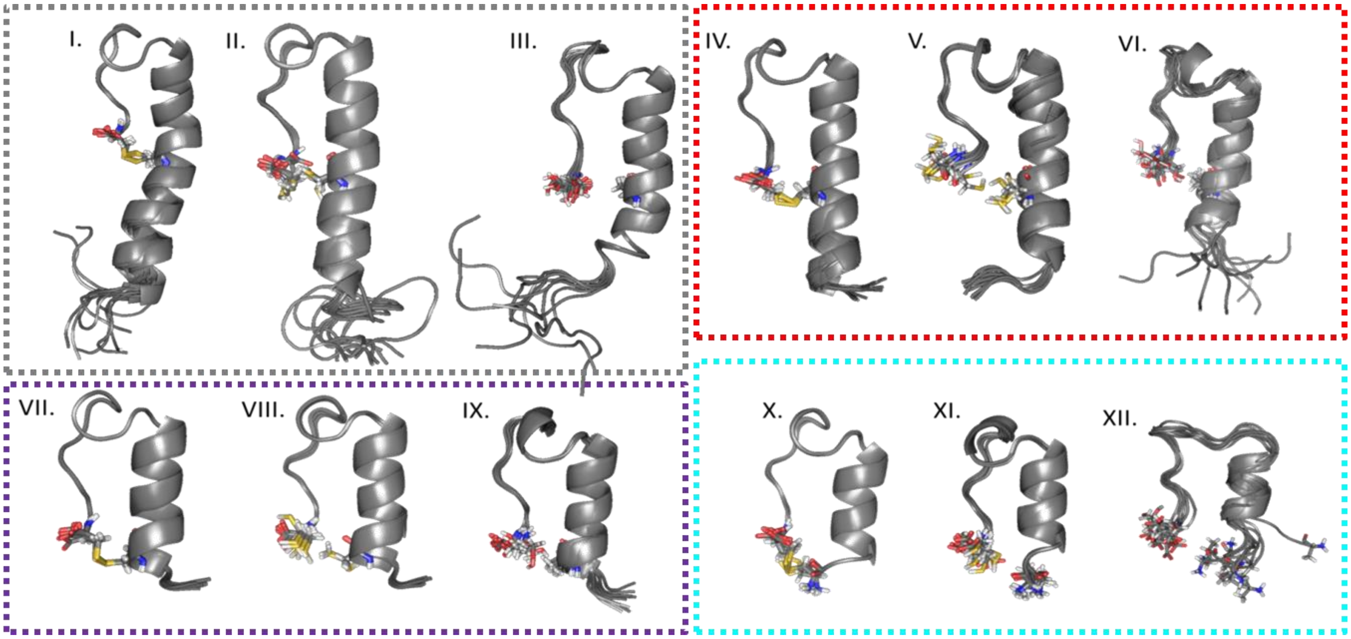

Supplementary Figure 2: The best 10 membered structure ensembles of our set of model proteins: I. E19_SS; II. E19_2SH; III. E19; IV. E11_SS; V. E11_2SH; VI. E11; VII. E5_SS; VIII. E5_2SH; IX. E5; X. E2_SS; XI. E2_2SH; XII. E2; The structure ensembles were determined by NOESY restraint sets at 15 °C *pH* = 7. For the number of assigned cross-peaks see **Stable 1.** The structure of the parent proteins (E19, E11, E5, E2) were determined by Rovó et al. [56]

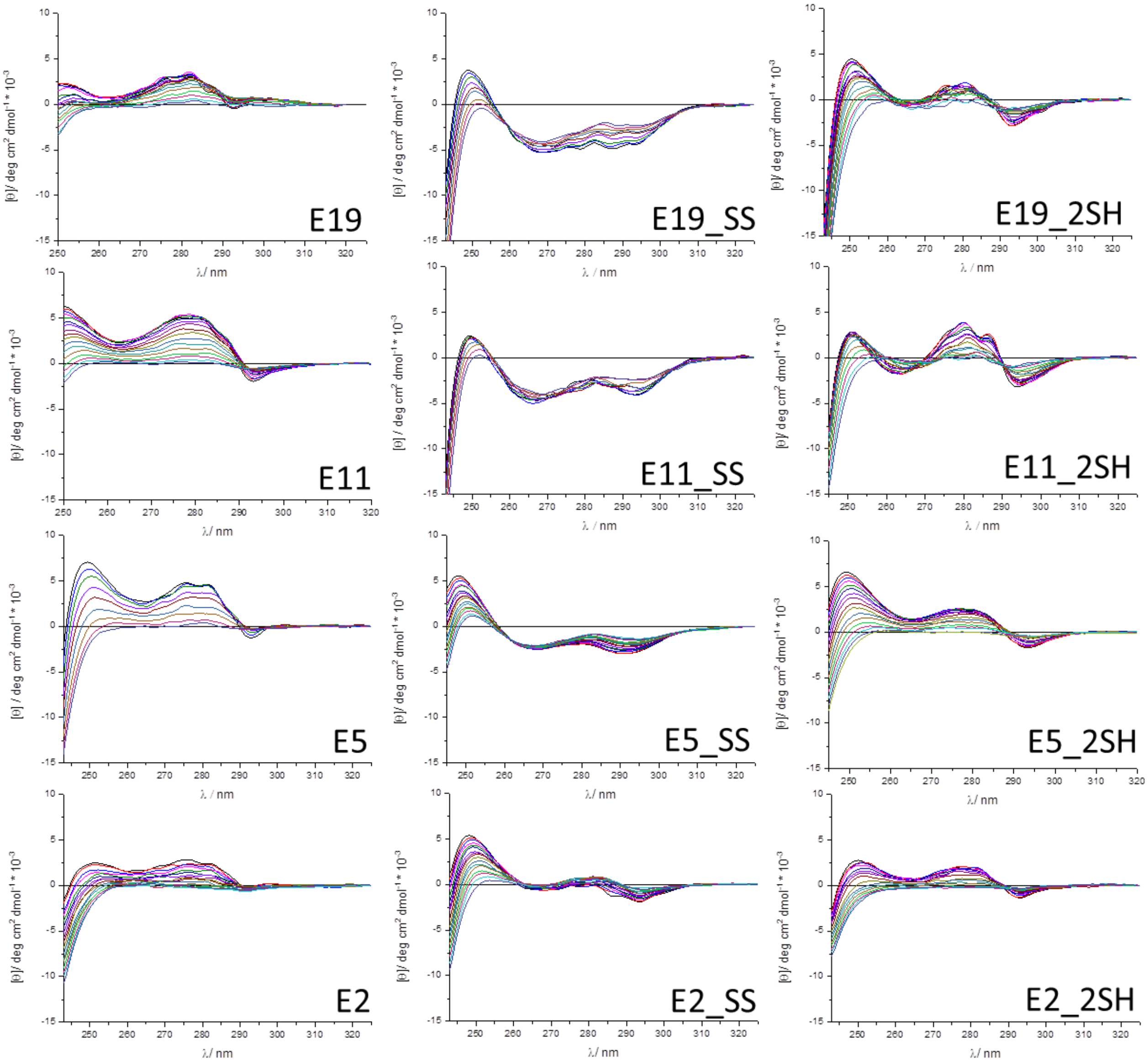

Supplementary Figure 3. Temperature dependent (5–85°C) near-UV ECD spectra (240–325 nm) of parent, SS-bond enhanced and SS-bond reduced Exenatide derivates.

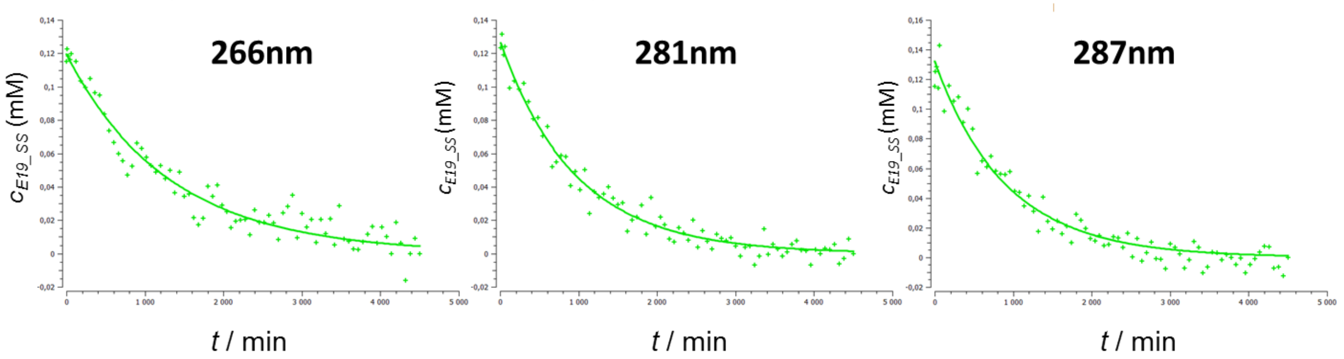

λ [nm]	k_1 reduction [L mmol $^{-1}$ min^{-1}]	stdev (k_1)	int. hw	upper limit	lower limit	$[SS]_0$ estimated [mM]	stdev ($[SS]_0$)	int. hw	upper limit	lower limit
266	4.11×10^{-4}	1.15×10^{-5}	2.63×10^{-5}	3.85×10^{-4}	4.37×10^{-4}	0.119	0.0020	0.0046	0.1149	0.1240
281	5.67×10^{-4}	1.25×10^{-5}	2.86×10^{-5}	5.38×10^{-4}	5.95×10^{-4}	0.127	0.0017	0.0039	0.1234	0.1313
287	5.98×10^{-4}	1.68×10^{-5}	3.85×10^{-5}	5.59×10^{-4}	6.36×10^{-4}	0.132	0.0022	0.0049	0.1272	0.1370

Supplementary Figure 4. Parameter estimation of the E19_SS reduction kinetics (~ 0.113 mM E19_SS, *pH* = 7, 15 °C, 18-fold excess of TCEP) monitored by near-UV ECD at 3 characteristic wavelengths, namely at 266, 281 and 287 nm. Stdev means standard deviation, int. hw is for the half-width of the 95% confidence interval whose upper and lower limit is also given.

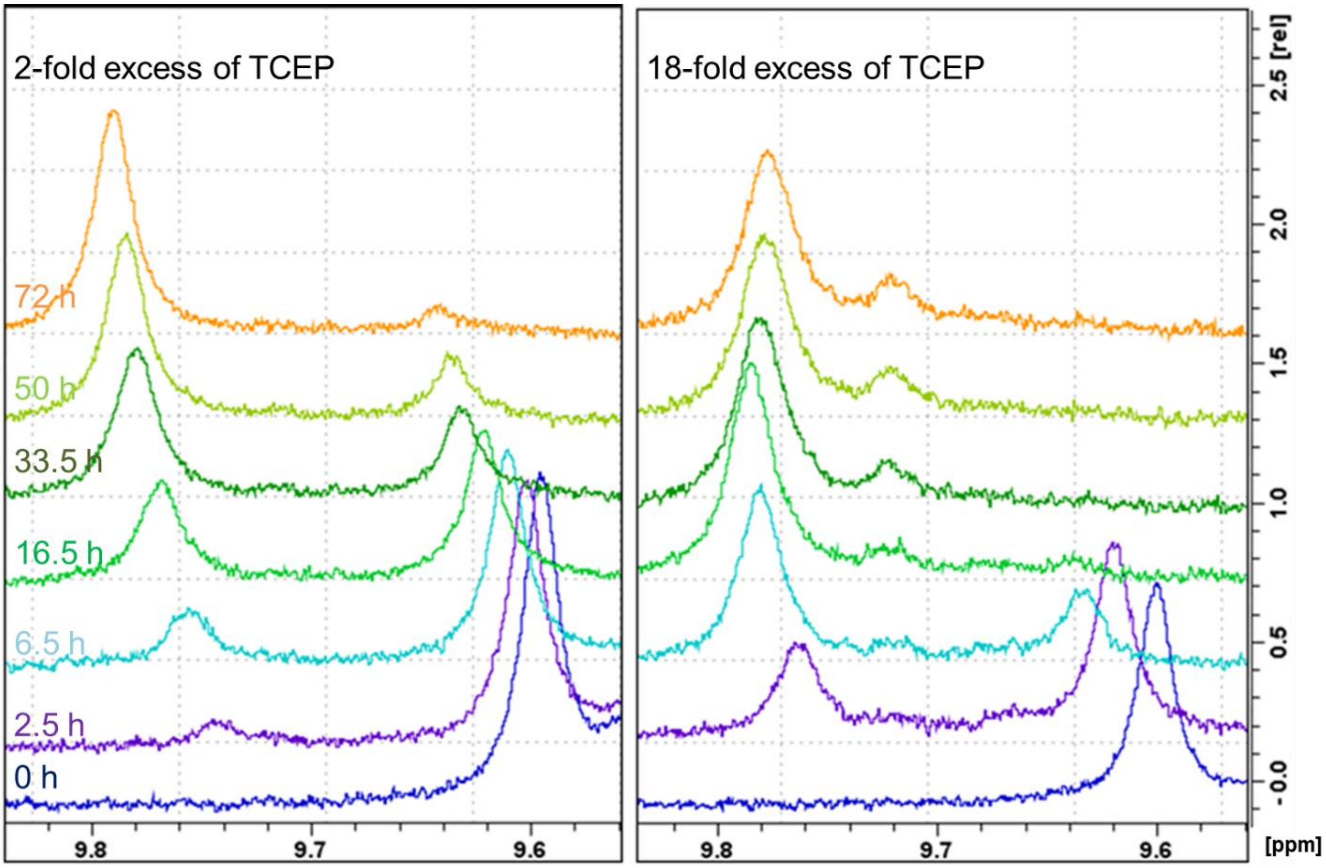

Supplementary Figure 5. SS-bond reduction monitored by the Hε1 (25Trp) resonance shift of E19_SS/E19_2SH at 2- and 18-fold molar excess of TCEP. The Hε1 resonance of the oxidized form is at 9.60 ppm with intensity / integral decreasing, while that of the reduced form (9.76 ppm) emerges as the reduction time proceeds. In the presence of a large excess of TCEP, an uncharacterized Hε1 resonance form appears (9.72 ppm), likely that of a by-product of the lengthy reduction.

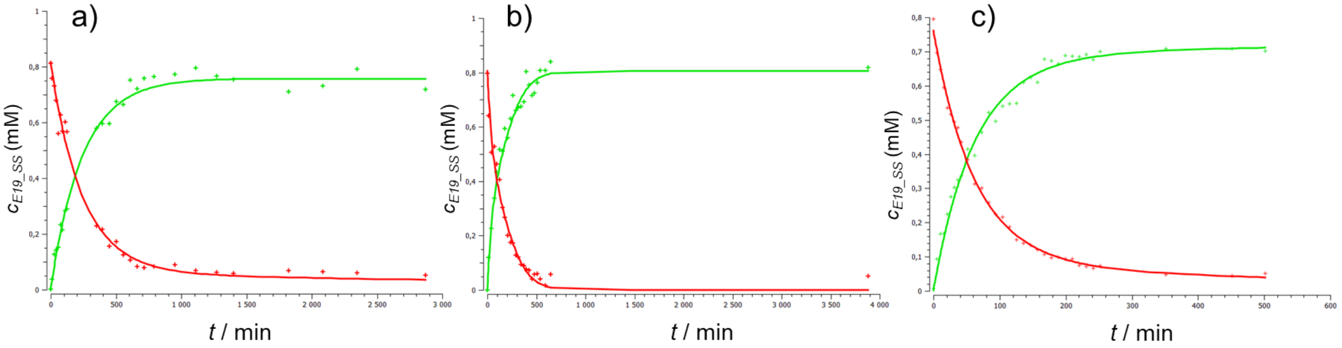

	experimental conditions						parameters								
E19_SS	T [C°]	[SS] [mM]	[RA_{red}] [mM]	[RA_{red}]/[SS]	Conversion rate [%]	elapsed time to steady state [h]	[O_2] [mM]	[SS] [mM]	Reduction k_1 [L mmol $^{-1}$ min^{-1}] Value	$t_{½}$ [min]	relative std. deviation [%]	Oxidation k_2 [L mmol $^{-1}$ min^{-1}] Value	relative std. deviation [%]	Precipitation k_3 [L mmol $^{-1}$ min^{-1}] Value	relative std. deviation [%]
a)	**15**	0.81	12.78	15.72	92	~15	1.00	0.82	3.05×10^{-4}	181	3.27	2.69×10^{-4}	107.40	1.30×10^{-5}	88.29
b)	**25**	0.80	12.78	16.00	94	~6	0.35	0.81	7.68×10^{-4}	72	9.78	1.86×10^{-2}	13.30	n.d.	n.d.
c)	**37**	0.80	12.78	15.95	94	~4	0.58	0.76	1.15×10^{-3}	48	1.68	2.27×10^{-3}	48.32	3.95×10^{-5}	109.24
–	**60**	0.81	12.78	15.75	n.d.	n.d.	n.d.	n.d.	n.d.	n.d.	n.d.	n.d.	n.d.	n.d.	n.d.

Supplementary Figure 6. Parameter estimation of the temperature dependent reduction of E19_SS (oxidized: red; reduced: green, by using 0.8 mM protein concentration and 18-fold excess of TCEP **a**) at 15 °C **b**) 25 °C **c**) 37 °C.

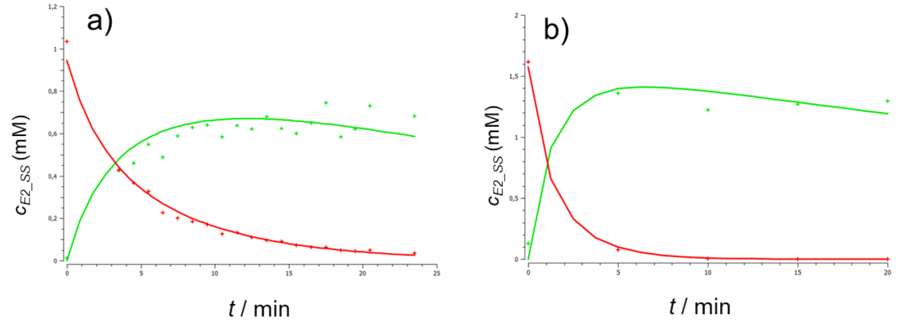

E2_SS	experimental conditions						parameters								
	T [C°]	[SS] [mM]	$[RA_{red}]$ [mM]	$[RA_{red}]$/[SS]	Conversion rate [%]	elapsed time to steady state [h]	$[O_2]$ [mM]	[SS] [mM]	Reduction k_1 [L mmol $^{-1}$ min^{-1}] Value	$t_{½}$ [min]	relative std. deviation [%]	Oxidation k_2 [L mmol $^{-1}$ min^{-1}] Value	relative std. deviation [%]	Precipitation k_3 [L mmol $^{-1}$ min^{-1}] Value	relative std. deviation [%]
a)	15	1.05	1.97	1.88	100	~15 m	0.06	0.94	1.37×10^{-1}	3	16.92	3.95×10^{-1}	117.83	2.31×10^{-3}	9.47
b)	15	1.74	3.26	1.87	100	$<$5 m	n.d.	1.57	2.59×10^{-1}	~1	15.37	n.d.	n.d.	1.52×10^{-2}	18.07

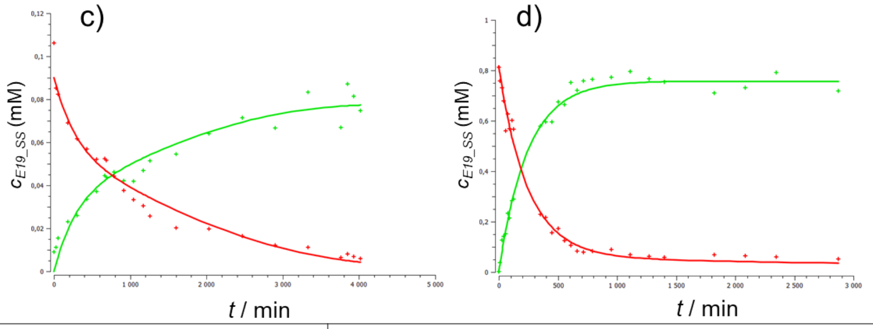

E19_SS	experimental conditions						parameters								
	T [C°]	[SS] [mM]	$[RA_{red}]$ [mM]	$[RA_{red}]$/[SS]	Conversion rate [%]	elapsed time to steady state [h]	$[O_2]$ [mM]	[SS] [mM]	Reduction k_1 [L mmol $^{-1}$ min^{-1}] Value	$t_{½}$ [min]	relative std. deviation [%]	Oxidation k_2 [L mmol $^{-1}$ min^{-1}] Value	relative std. deviation [%]	Precipitation k_3 [L mmol $^{-1}$ min^{-1}] Value	relative std. deviation [%]
c)	15	0.12	1.88	16.29	92	~40	0.07	0.09	8.03×10^{-4}	466	12.37	1.72×10^{-2}	14.77	3.60×10^{-5}	36.17
d)	15	0.81	12.78	15.72	92	~15	1.00	0.82	3.05×10^{-4}	181	3.27	2.69×10^{-4}	107.40	1.30×10^{-5}	88.29

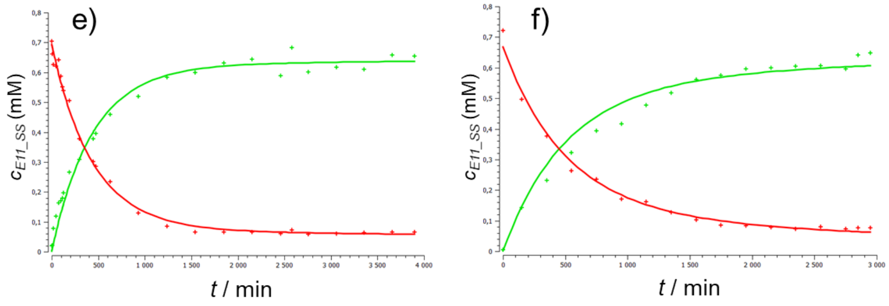

E11_SS	experimental conditions						parameters								
	T [C°]	[SS] [mM]	$[RA_{red}]$ [mM]	$[RA_{red}]$/[SS]	Conversion rate [%]	elapsed time to steady state [h]	$[O_2]$ [mM]	[SS] [mM]	Reduction k_1 [L mmol $^{-1}$ min^{-1}] Value	$t_{½}$ [min]	relative std. deviation [%]	Oxidation k_2 [L mmol $^{-1}$ min^{-1}] Value	relative std. deviation [%]	Precipitation k_3 [L mmol $^{-1}$ min^{-1}] Valu e	relative std. deviation [%]
e)	15	0.73	12.78	17.61	91	~28	2.00	0.69	1.64×10^{-4}	337	2.98	1.07×10^{-4}	154.66	n.d.	n.d.
f)	15	0.73	6.47	8.90	89	~35	0.50	0.67	2.61×10^{-4}	423	4.80	4.57×10^{-4}	72.94	n.d.	n.d.

Supplementary Figure 7. SS-bond reduction as a bimolecular nucleophilic substitution (S_N2) reaction both the concentration of the initial oxidized protein (oxidized: red; reduced: green). and the reagent influences the reduction rate. To quantify this extent the initial peptide concentrations were altered for the shortest E2_SS (a,b) and the longest E19_SS(c,d) reductions. The experiments were carried out at 15 °C, for E2_SS reductions ~ 2-fold excess of TCEP were used while in case of E19_SS ~16-fold excess of TCEP. In case of E11_SS (e,f) the excess of TCEP was altered, while concentration of E11_SS was set to 0.73 mM and the temperature to 15 °C.

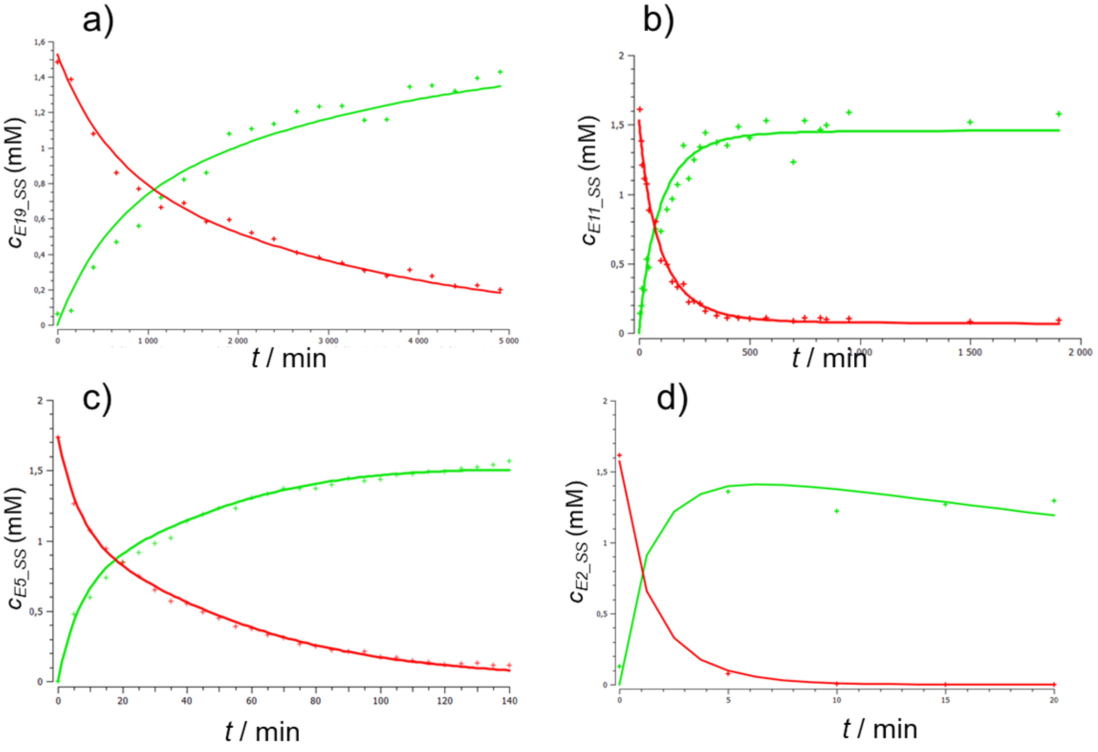

		experimental conditions						parameters								
		T [C°]	[SS] [mM]	$[RA_{red}]$ [mM]	$[RA_{red}]$/[SS]	Conversion rate [%]	elapsed time to steady state [h]	$[O_2]$ [mM]	[SS] [mM]	Reduction k_1 [L mmol $^{-1}$ min^{-1}]			Oxidation k_2 [L mmol $^{-1}$ min^{-1}]		Precipitation k_3 [L mmol $^{-1}$ min^{-1}]	
										Value	$t_{½}$ [min]	relative std. deviation [%]	Value	relative std. deviation [%]	Value	relative std. deviation [%]
a)	E19_SS	15	1.55	3.26	2.10	87	~76 h	0.33	1.53	2.71×10^{-4}	909	7.30	7.24×10^{-4}	26.96	n.d.	n.d.
b)	E11_SS	15	1.72	3.26	1.89	94	~5–6 h	1.80	1.56	3.68×10^{-3}	67	2.61	1.00×10^{-6}	14.66	1.00×10^{-6}	249.10
c)	E5_SS	15	1.74	3.26	1.88	93	~1 h	0.58	1.70	1.85×10^{-2}	14	3.35	4.53×10^{-2}	5.71	5.57×10^{-4}	16.47
d)	E2_SS	15	1.74	3.26	1.87	100	<5 m	n.d.	1.57	2.59×10^{-1}	~1	15.37	n.d.	n.d.	1.52×10^{-2}	18.07

Supplementary Figure 8. Estimated kinetic parameters of the different length E19_SS variants: **a**) E19_SS, **b**) E11_SS **c**) E5_SS **d**) E2_SS (oxidized: red; reduced: green). For each reduction, 1.7 mM protein concentration was used with 2-fold excess of TCEP at 15 °C. Back-oxidation was ignored for E2_SS, due to the fast reduction rate and the short-term of observation period.

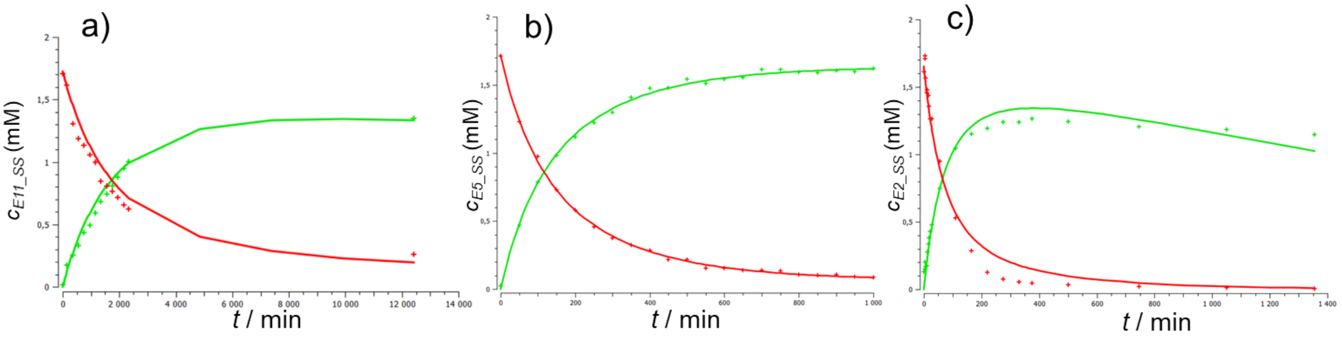

	experimental conditions						parameters									
	T [C°]	[SS] [mM]	$[RA_{red}]$ [mM]	$[RA_{red}]/[SS]$	Conversion rate [%]	elapsed time to steady state [h]	$[O_2]$ [mM]	[SS] [mM]	Reduction k_1 [L mmol $^{-1}$ min^{-1}] Value	$t_{½}$ [min]	relative std. deviation [%]	Oxidation k_2 [L mmol $^{-1}$ min^{-1}] Value	relative std. deviation [%]	Precipitation k_3 [L mmol $^{-1}$ min^{-1}] Value	relative std. deviation [%]
a) E11_SS	15	1.72	3.26	1.89	~84	138 h	0.024	n.d.	1.52×10^{-4}	1659	45.152	5.06×10^{-4}	7154.71	3.86×10^{-5}	20.686
b) E5_SS	15	1.74	3.26	1.88	95	9–10 h	2.000	1.72	2.18×10^{-3}	115	1.064	6.38×10^{-5}	437.15	1.14×10^{-5}	80.208
c) E2_SS	15	1.74	3.26	1.87	100	5h	0.334	1.65	4.04×10^{-3}	62	5.315	2.21×10^{-3}	98.22	3.87×10^{-4}	9.881

Supplementary Figure 9. Estimated kinetic parameters of the different length E19_SS variants: **a**) E11_SS **b)** E5_SS **c**) E2_SS (oxidized: red; reduced: green). For each reduction, 1.7 mM protein concentration was used with 2-fold excess of DTT at 15 °C.

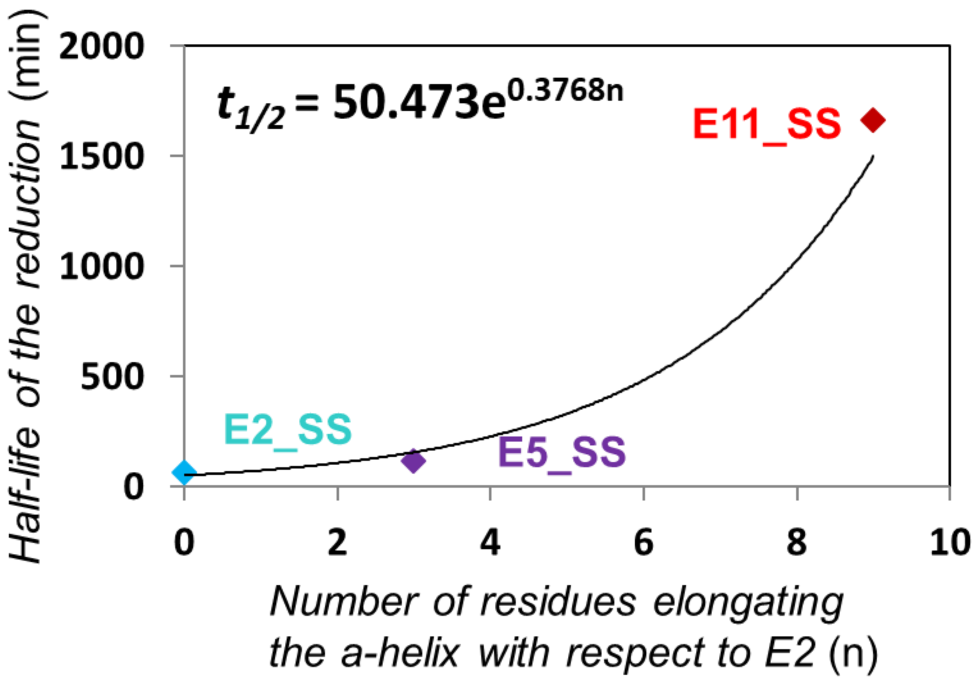

Supplementary Figure 10. Dependence of the reduction half-life on the helix length when using 2-fold excess of DTT as reducing agent with 1.7 mM protein at 15 °C.

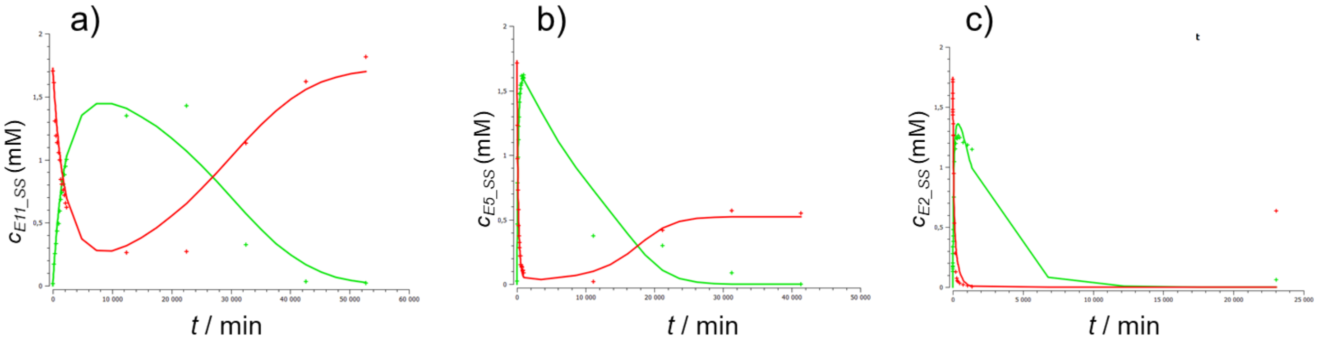

		$[O_2]$ [mM]	[SS] [mM]	Reduction k_1 [L mmol $^{-1}$ min^{-1}]	relative std. deviation [%]	Oxidation k_2 [L mmol $^{-1}$ min^{-1}]	relative std. deviation [%]	Precipitation k_3 [L mmol $^{-1}$ min^{-1}]	relative std. deviation [%]	O_2 diffusion k_4 [L mmol $^{-1}$ min^{-1}]	relative std. deviation [%]
a)	E11_SS	1.00×10^{-6}	1.65	1.54×10^{-4}	9.3	1.83×10^{-5}	760.3	1.00×10^{-8}	21071.7	3.20×10^{-4}	538.1
b)	E5_SS	1.00	1.73	2.11×10^{-3}	2.5	3.12×10^{-5}	5657.6	7.11×10^{-5}	6.4	4.75×10^{-4}	4441.9
c)	E2_SS*	4.34×10^{-1}	1.72	4.29×10^{-3}	7.8	2.97×10^{-3}	91.9	4.65×10^{-4}	10.6	n.d.	n.d.

Supplementary Figure 11: Estimated kinetic parameters of the different length E19_SS variants: **a**) E11_SS **b**) E5_SS **c**) E2_SS (oxidized: red; reduced: green). For each reduction, 1.7 mM protein concentration was used with 2-fold excess of DTT at 15 °C. *Due to the inappropriate sampling parameters of E2_SS the redox-cycle here cannot be unambiguously estimated, nevertheless the last measured point proves that both re-oxidation and oligomerization (precipitation) occur.

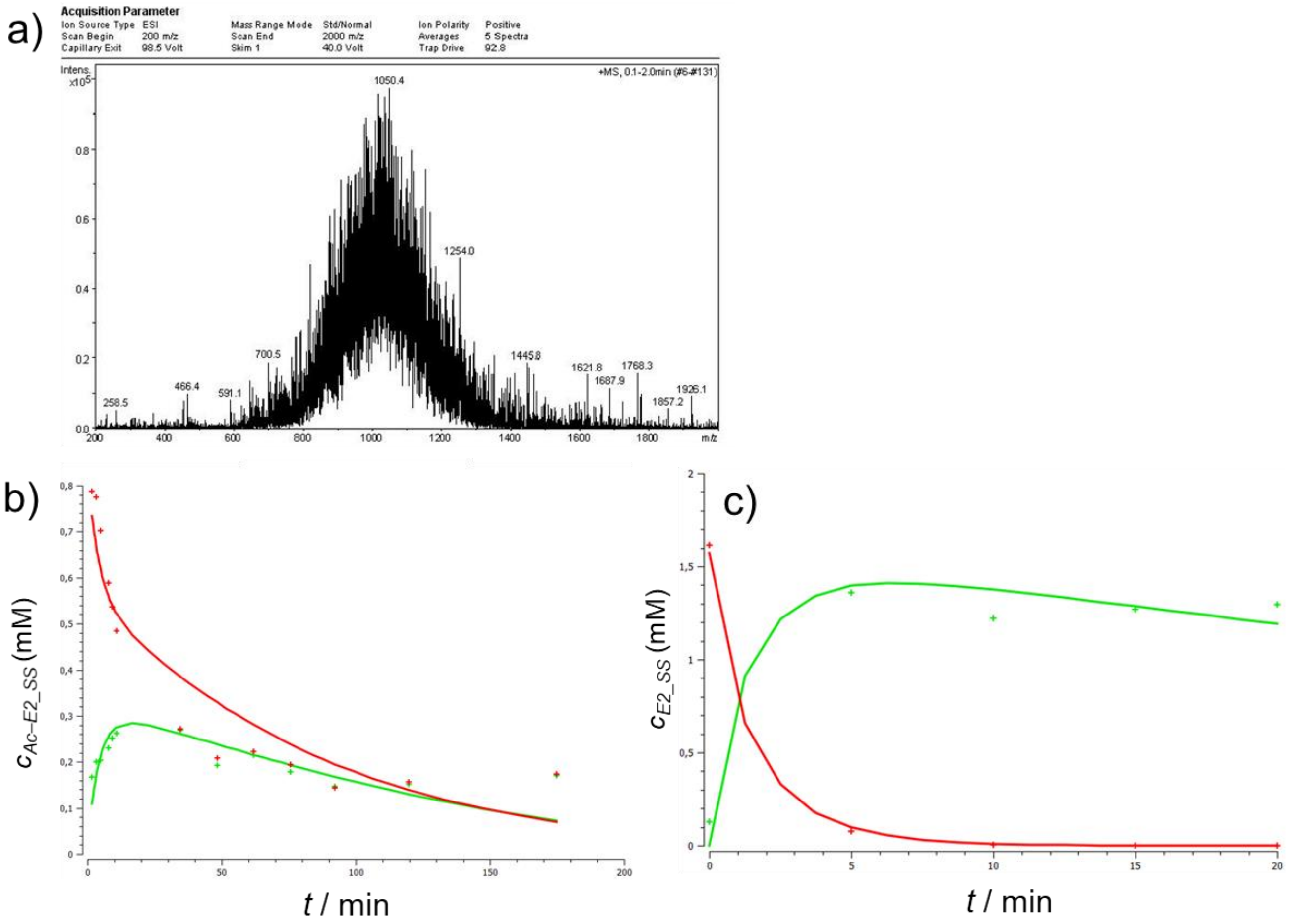

		$[O_2]$ [mM]	[SS] [mM]	Reduction k_1 [L mmol $^{-1}$ min^{-1}]	relative std. deviation [%]	Oxidation k_2 [L mmol $^{-1}$ min^{-1}]	relative std. deviation [%]	Precipitation k_3 [L mmol $^{-1}$ min^{-1}]	relative std. deviation [%]
b)	Ac–E2_SS	1.71	0.84	2.79×10^{-2}	15.9	7.17×10^{-2}	34.7	2.30×10^{-2}	9.9
c)	E2_SS	n.d.	1.575	2.59×10^{-1}	15.4	n.d.	n.d.	1.52×10^{-2}	18.1

Supplementary Figure 12. a) ESI-MS spectra of the heterogeneous aggregate of Ac-E2_SS after 2000 minutes reaction time followed by a HPLC purification. **b**) Fit of the kinetic model of Ac-E2_SS. Crosses show the measured data, while continuous curves show the fitted model. Red color is for the (oxidized) SS species, while green color is for the (reduced) 2SH species. For the reduction, ~ 1.7 mM initial Ac-E2_SS was used with 2-fold excess of TCEP at 15 °C and *pH* = 7. During the time between the first measured point and the addition of the reducing agent (~ 3 minutes) almost half of the dissolved protein precipitated. That is why the parameter estimation begins from 0.84 mM of Ac-E2_SS. **c)** For the reduction, ~ 1.7 mM initial E2_SS was used with 2-fold excess of TCEP at 15 °C and *pH* = 7. On this short timescale (20 min) the back oxidation was negligible (scale of 10^{-6}) therefore it wasn't involved in parameter estimation.

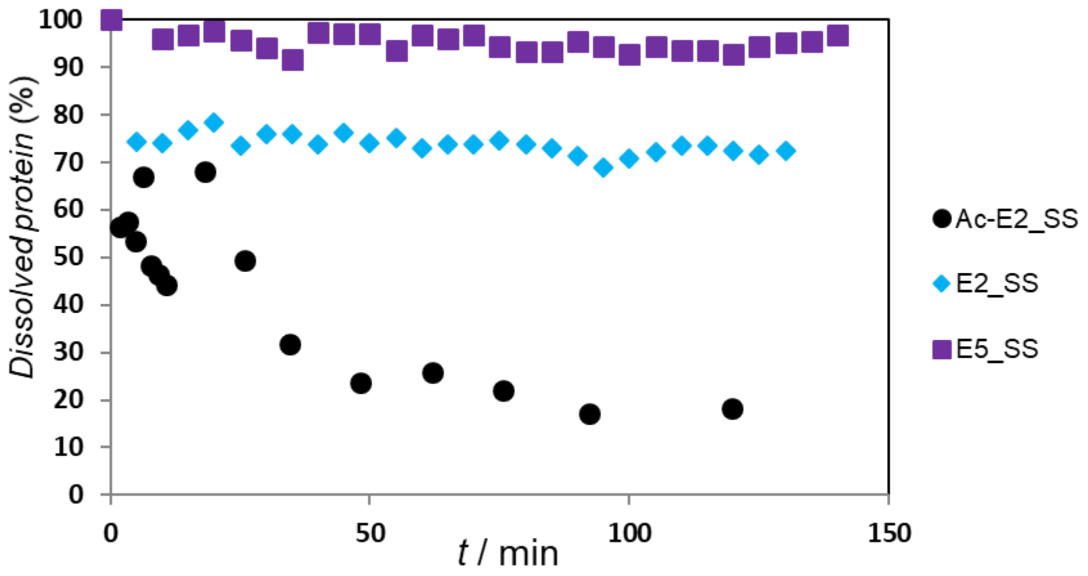

Supplementary Figure 13. Dissolved protein ratio (in %) of E5_SS, E2_SS and Ac_E2_SS compared to their starting values (before adding the TCEP) as a function of time. In each of the cases, ~1.7 mM protein concentration was used with 2-fold excess of TCEP at 15 °C and *pH* = 7. Ac-E2_SS almost immediately precipitates right at the beginning of the “short” observation period of time.

		i,i	i,i+1	i,i+2	i,i+3	i,i+4	i,i+n,n>5	Sum	RMSD backbone	RMSD all atom
I.	E19_SS	456	219	32	109	48	119	983	0.29	0.60
II.	E19_2SH	428	157	23	78	36	91	813	0.45	0.72
III.	E19	284	185	26	114	78	87	774	0.33	1.15
IV.	E11_SS	376	149	22	85	36	113	781	0.36	0.67
V.	E11_2SH	372	146	20	59	39	105	741	0.43	0.76
VI.	E11	174	61	3	46	21	53	358	1.37	2.28
VII.	E5_SS	274	136	17	53	28	101	609	0.56	1.24
VIII.	E5_2SH	264	123	28	59	25	81	580	0.62	1.35
IX.	E5	176	100	17	29	15	41	378	0.58	2.21
X.	E2_SS	259	143	36	64	30	124	656	0.11	0.44
XI.	E2_2SH	241	98	26	32	15	65	477	0.50	0.91
XII.	E2	136	37	2	4	5	19	203	1.24	2.16

Supplementary Table 1. The NOE derived restraint sets applied for the 10 membered structure ensemble calculations (Supplementary Figure 2. I-XII.). Restraints were categorized from intraresidual (i,i) to long range (i,i+n) ones, while structures with their RMSD values.
